# The MCM-Binding Protein ETG1 Aids Sister Chromatid Cohesion Required for Postreplicative Homologous Recombination Repair

**DOI:** 10.1371/journal.pgen.1000817

**Published:** 2010-01-15

**Authors:** Naoki Takahashi, Mauricio Quimbaya, Veit Schubert, Tim Lammens, Klaas Vandepoele, Ingo Schubert, Minami Matsui, Dirk Inzé, Geert Berx, Lieven De Veylder

**Affiliations:** 1 Department of Plant Systems Biology, Flanders Institute for Biotechnology (VIB), Gent, Belgium; 2 Department of Plant Biotechnology and Genetics, Ghent University, Gent, Belgium; 3 Plant Functional Genomics Research Group, RIKEN Plant Science Center, Yokohama, Kanagawa, Japan; 4 Department for Molecular Biomedical Research, Molecular and Cellular Oncology Unit, Flanders Institute for Biotechnology (VIB), Gent, Belgium; 5 Department of Biomedical Molecular Biology, Ghent University, Gent, Belgium; 6 Leibniz Institute of Plant Genetics and Crop Plant Research (IPK), Gatersleben, Germany; Institut Jean-Pierre Bourgin, INRA de Versailles, France

## Abstract

The DNA replication process represents a source of DNA stress that causes potentially spontaneous genome damage. This effect might be strengthened by mutations in crucial replication factors, requiring the activation of DNA damage checkpoints to enable DNA repair before anaphase onset. Here, we demonstrate that depletion of the evolutionarily conserved minichromosome maintenance helicase-binding protein ETG1 of *Arabidopsis thaliana* resulted in a stringent late G2 cell cycle arrest. This arrest correlated with a partial loss of sister chromatid cohesion. The lack-of-cohesion phenotype was intensified in plants without functional CTF18, a replication fork factor needed for cohesion establishment. The synergistic effect of the *etg1* and *ctf18* mutants on sister chromatid cohesion strengthened the impact on plant growth of the replication stress caused by *ETG1* deficiency because of inefficient DNA repair. We conclude that the ETG1 replication factor is required for efficient cohesion and that cohesion establishment is essential for proper development of plants suffering from endogenous DNA stress. Cohesion defects observed upon knockdown of its human counterpart suggest an equally important developmental role for the orthologous mammalian ETG1 protein.

## Author Summary

DNA replication is a highly complex process and the source of potential DNA damage. It is of utmost importance that the damaged DNA is repaired before cells proceed through mitosis, because the genome holds all the information required for correct development. DNA replication results in two identical sister chromatids. A trick applied by cells to overcome damaged DNA is homologous recombination, using the undamaged copy of the sister chromatid as a template to repair the damaged one. This process is aided by keeping the two sister chromatids in close proximity after the replication process by the deposition of a molecular glue, called cohesin. In the present work, we identified the *Arabidopsis thaliana* ETG1 protein as a novel evolutionarily conserved replication factor that is needed for maintaining the sister chromatids physically aligned. In plants without ETG1, DNA damage builds up due to inefficient DNA repair. As a consequence, cell division is impaired with a huge impact on plant growth, highlighting the importance of cohesin for the correct development of eukaryotic organisms. Cohesion phenotypes observed upon the depletion of the orthologous human ETG1 protein indicate equally prominent roles for this particular factor during mammalian development.

## Introduction

For one single cell to generate two cells, numerous events must be coordinated, in particular, faithful DNA replication and partitioning of the sister chromatids to each of the daughter cells. It is of utmost importance to ensure the error-free duplication of the replicated DNA during each cell cycle, preventing the transmission of potentially harmful mutations to the daughter cells, which otherwise might result in developmental defects or even cancer. DNA damage and replication errors might originate from DNA stress provoked by either exogenous (such as γ-irradiation and UV-B light) or endogenous (such as metabolic byproducts) sources. The latter include the replication process itself that necessitates the cooperation of many different proteins in a highly complex manner. Errors arisen during replication are preferentially repaired through homologous recombination between the replicated sister chromatids that lay in close proximity thanks to cohesion. This sister chromatid cohesion is mediated by cohesin that consists of four subunits in budding yeast (*Saccharomyces cerevisiae*): two structural maintenance of chromosome (SMC) proteins, designated SMC1 and SMC3, and two non-SMC subunits, designated SCC1 (also known as Mcd1/Rad21) and SCC3 [1]–[5]. SMC1 and SMC3 are self-folded by antiparallel coiled-coil interactions, creating a rod-shaped molecule with an ATP-binding “head” at one end and a “hinge” domain at the other. The two SMC subunits associate with each other through their hinge domains, producing a V-shaped dimer [6],[7]. Cohesin forms a tripartite ring in which the open-V structure of the SMC heterodimer is closed by the simultaneous binding of the N- and C-terminal regions of SCC1 to the head domains of SMC3 and SMC1, respectively [8].

Cohesin is deposited on unreplicated chromatin in a reaction requiring ATP hydrolysis by the SMC heads and the cohesin-loading complex SCC2/SCC4 [9]–[11]. Whereas cohesion loading occurs well before S phase, the process of cohesion establishment is intimately connected with DNA replication. In budding yeast, cohesion depends on an acetyltransferase, designated Eco1/Ctf7, that acetylates two lysine residues on the ATPase head domain of SMC3 [12]–[14]. Eco1/Ctf7 interacts physically and genetically with the proliferating cell nuclear antigen (PCNA) and the replication factor C (RFC) and has been found to travel along the DNA with replication forks [15]–[17], suggesting that replication fork progression and sister chromatid cohesion are coupled events. This model is supported by the observation that cohesion defects are caused by mutations in replisome components, e.g., the DNA polymerase α-binding protein Ctf4 [18]–[19], the Chl1 helicase [20], and RFC components, such as Ctf18 [21]. In budding yeast and humans, Ctf18 associates with Rfc2, Rfc3, Rfc4, and Rfc5 to form the RFC^Ctf18^ complex, which, in turn, couples with two additional subunits, Dcc1 and Ctf8, creating a heptameric complex with the PCNA that has a loading and unloading activity and plays a role in sister chromatid cohesion [19]–[23].

E2F transcription factors control the expression of many genes involved in DNA replication and DNA repair. By studying *Arabidopsis thaliana* E2F target genes, we have previously identified the E2F TARGET GENE 1 (ETG1) as a novel evolutionarily conserved replisome factor. ETG1 binds with the minichromosome maintenance (MCM) complex and is crucial for efficient DNA replication [24]. Similarly to ETG1, the human orthologous MCM-BP protein binds to replication origins as part of the MCM complex [25]. Plants lacking the *ETG1* gene have serrated leaves because of cell cycle inhibition triggered by the DNA replication checkpoints, as shown by the transcriptional induction of DNA stress and checkpoint genes [24]. Here we demonstrate that the ETG1 protein plays an additional role in the establishment of sister chromatid cohesion. Cohesion along chromosome arms was impaired in *ETG1*-deficient cells. Strikingly, the growth inhibition and the DNA stress phenotype of *etg1* mutant plants were strongly enhanced in cohesion mutant backgrounds. Decreased DNA repair kinetics illustrate that the lack of cohesion accounts for severe growth defects in plants suffering from endogenous DNA stress, emphasizing the importance of cohesion for correct plant development. Finally, we show that the knockdown of the human *MCM-BP* results in a loss of cohesion phenotype as well, suggesting an equally important developmental role for the orthologous mammalian ETG1 proteins.

## Results

### Upregulation of mitosis-specific genes in *etg1* mutants

**Figure 1:**
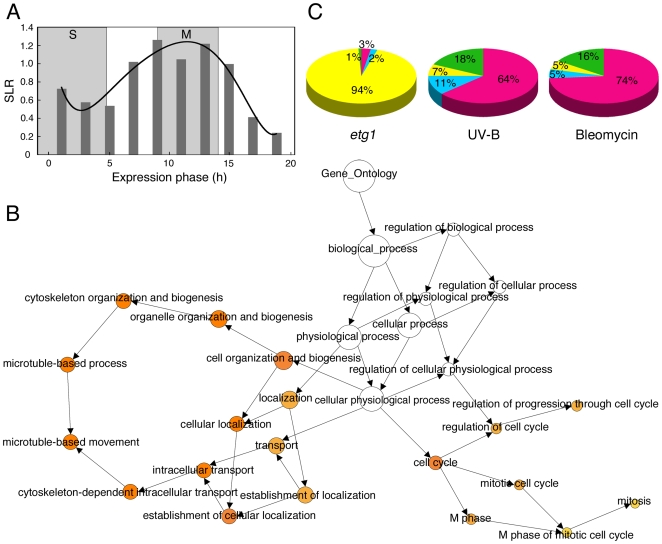
Upregulation of G2/M-specific genes in *ETG1*-deficient plants. (A) Plot of average signal log ratios (SLRs; logarithms to the base 2) between wild-type and *etg1* mutant plants for 9,910 genes during the 22-h cell cycle of *Arabidopsis* [26]. Genes were sorted into 10 bins representing the 10 time points of measurements, based on their maximal expression during the cell cycle. Cells were synchronized by a release from an aphidicolin arrest. Synchronized cells completed S phase in 5 h and went through mitosis from 9 to 14 h. The solid line represents the best-fitted curve. (B) GO analysis of the 196 upregulated genes in the first leaves of *etg1* mutant plants. The yellow-to-orange color of the circles correspond to the level of significance of the overrepresented GO category of ≤0.01 according to a multiple *t* test with false discovery rate–corrected P value. The size of the circle is proportional to the number of genes in the category. (C) Comparison of the distribution of cell cycle phase-dependent upregulated genes in *etg1* plants and plants treated with UV-B- or bleomycin. Microarray data sets of UV-B, and bleomycin treatment were imported from [28] and [29], respectively. S (red), G2 (blue), M (yellow), and G1 (green) phase-specific gene expression patterns were defined by Menges et al. [26].

*ETG1*-deficient plants suffer from endogenous DNA stress and display a transient cell cycle arrest [24]. To gain more insight into this defective cell cycle, we examined transcript levels of 22,750 genes by using Affymetrix ATH1 GeneChip arrays. Triplicate batches of proliferating first leaves of 9-day-old wild-type and two independent *etg1-1* and *etg1-2* mutant plants were harvested for total RNA preparation. Statistical analysis identified a total of 219 genes differentially expressed between wild-type and *etg1* plants at a P-value <0.01, among which 89% upregulated and 11% downregulated and displaying a 1.3- to 14.8-fold change in expression. Strikingly, of the 195 upregulated genes, 103 (53%) showed an expression peak during mitosis (Table S1 and Table S2; Figure S1). Transcription of genes expressed specifically during mitosis is regulated by a common upstream *cis*-acting element (ynCAACGG), designated mitosis-specific activator (MSA). In total, 82 upregulated genes in *etg1* plants possessed an MSA element within the first 1 kb region upstream of the translation start, which is significantly more than expected by chance (P-value <0.001) and indicative for an arrest in late G2 or mitosis. This hypothesis was corroborated by an overall transcriptional induction of G2 and M-phase expressed genes in *etg1* knockout plants, as demonstrated by plotting the average signal log ratios (SLRs) between the expression levels in wild-type and *etg1* mutant plants of 9,910 genes previously defined as cell cycle regulated [26] (Figure 1A).

To identify the biological processes that might be affected in the *etg1* mutant plants, the up- and downregulated genes were analyzed for gene ontology (GO) enrichment [27]. Among the upregulated genes, regulation of progression through cell cycle, mitotic cell cycle, and microtubule-based movement genes were significantly overrepresented (Figure 1B), indicating that lack of *ETG1* resulted in mitotic defects. This result was unexpected because *ETG1* is an E2F-dependent target gene expressed during the S phase and its gene product is required for DNA replication. Additionally, *ETG1*-deficient plants had been found to interact synergistically with the *wee1* and *atr1* replication checkpoint mutants and to suffer from DNA replication stress [24], which is anticipated to arrest the cell cycle during S or early G2. Indeed, when the *etg1* microarray data set was compared with that of plants exposed to UV-B light [28] or to the radiomimetic drug bleomycin [29], the distribution of the significantly upregulated cell cycle phase markers was different. In the latter cases, the set of modified genes was clearly enriched for S-phase genes (Figure 1C), suggesting that the cell cycle arrest in the *etg1* mutants occurs downstream of that one triggered by UV-B or bleomycin.

### ETG1 is required for sister chromatid arm cohesion

**Figure 2:**
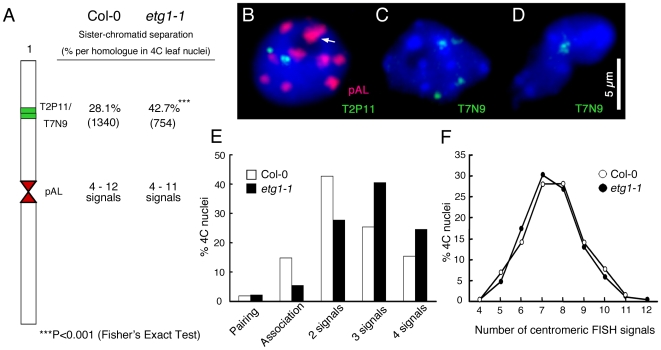
Requirement of ETG1 for establishment of sister chromatid arm cohesion. (A) Percentage of positional separation frequencies per homologous chromosome (number of investigated nuclei in parentheses) analyzed in wild-type (Col-0) and *etg1-1* mutant plants after FISH with the labeled T2P11 or T7N9 BACs from chromosome 1. The FISH probe pAL detects the centromeric 178-bp repeats. (B–D) Structural arrangement of FISH signal positions in 4C nuclei counterstained with DAPI. (B) Positional alignment (T2P11) at both chromosome 1 homologs. Two of 10 centromeric signals associated (arrow). (C) Positional sister chromatid separation at both homologs. (D) Positional association of both homologs. (E) Percentage of sister chromatid alignment/separation frequencies analyzed in wild-type (Col-0) and *etg1-1*. (F) Identical frequencies of centromere-specific FISH signals in wild-type and *etg1-1* nuclei.

Sister chromatid cohesion is essential for the appropriate distribution of chromosomes into the daughter cells upon cell division. Several observations indicate that cohesion establishment is coupled with DNA replication [18]–[21]. Therefore, we analyzed by fluorescent in situ hybridization (FISH) whether the cell cycle arrest in *etg1* mutant plants might be linked with a loss of cohesion. The two adjacent overlapping bacterial artificial chromosomes (BACs) T2P11 and T7N9 that label the upper mid-arm position of chromosome 1 were used for FISH analysis on flow-sorted 4C leaf nuclei to compare the frequency of sister arm alignment between the wild-type and *etg1-1*. The number of FISH signals indicates whether sister chromatids are linked at the position tested: one (pairing of both homologs) or two FISH signals mark the positional alignment of sister chromatids at the corresponding region and three or four signals the sister chromatid separation at one or both homologous chromosomes, respectively (Figure 2B–2D). When compared to the wild-type nuclei, the *etg1-1* nuclei showed a significant increase (P-value <0.001) in sister chromatid separation (Figure 2A and 2E). Positional sister chromatid separation occurred in 28.1% of the homologs in 4C wild-type leaf nuclei, whereas *etg1-1* leaf nuclei displayed 42.7% sister chromatid separation per homolog (Figure 2A). By contrast, FISH applied to the 178-bp centromere-specific sequence (pAL) revealed 4–12 signals in both the wild-type and the *etg1-1* mutant 4C nuclei (Figure 2A and 2F). These findings suggest that ETG1 is required for correct establishment of sister chromatid cohesion along chromosome arms, but not at centromeres.

### *Arabidopsis CTF18* is required for sister chromatid cohesion

**Figure 3:**
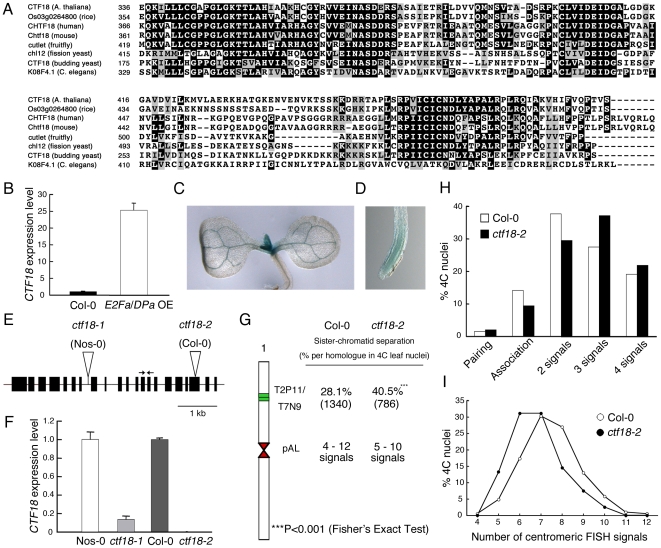
Importance of *Arabidopsis* CTF18 for sister chromatid cohesion. (A) Alignment of the AAA domain of CTF18 with its orthologous proteins: CHTF18 (NP_071375; human), Chtf18 (NP_663384; mouse), cutlet (NP_787969; fruitfly), Os03g0264800 (rice), CTF18 (NP_013795; budding yeast), chl12 (NP_595200; fission yeast), and K08F4.1 (NP_501841; *C. elegans*). (B) Real-time RT-PCR analysis of *CTF18* expression in wild-type (WT; Col-0) and *E2Fa*-*DPa*-overexpressing (E2Fa/DPa^OE^) plants. Total RNA prepared from 6-day-old plants was amplified by RT-PCR. All values were normalized against the expression level of the *ACTIN2* gene. (C,D) Histochemical localization of the GUS activity in transgenic 7-day-old shoot (C) and root (D) carrying the *CTF18* promoter. (E) Exon (boxes) and intron (lines) structure of *CTF18*. White triangles and arrows indicate T-DNA insertion sites and primer positions used for real-time RT-PCR analysis, respectively. (F) Real-time RT-PCR analysis of *CTF18* expression in wild-type (Nos-0 and Col-0), *ctf18-1*, and *ctf18-2* plants. Total RNA prepared from 9-day-old plants was amplified by RT-PCR. All values were normalized against the expression level of the *ACTIN2* gene. (G) Positional sister arm separation frequency detection by FISH detecting ∼100 kb mid-arm segments (BACs T2P11 and T7N9) and centromeric signal number detected by the 178-bp pAL probe. (H) Percentage of sister chromatid alignment/separation frequencies analyzed in wild-type (Col-0) and *ctf18-2* after FISH with labeled BACs T2P11 and T7N9 from chromosome 1. (I) Similar frequencies of centromere-specific FISH signals in wild-type and *ctf18-2* nuclei.

The *ETG1* gene had originally been identified by microarray analysis as a transcript induced in plants that ectopically expressed the heterodimeric *E2Fa-DPa* transcription factor [30] and had been demonstrated to be directly controlled by the E2Fa and E2Fb transcription factors [24]. Interestingly, putative orthologous genes encoding proteins involved in cohesion establishment during the replication process were also transcriptionally upregulated in *E2Fa-DPa*-overexpressing plants, such as *ECO1* (also known as *CTF7*) (At4g31400), *CHL1* (At1g79890), and *CTF18* (At1g04730) (Figure S2). In budding yeast, Ctf18 localizes at the replication fork and is a critical factor for establishment of cohesion [16],[19]. Putative orthologous proteins were found in human, mouse, Arabidopsis, rice, fruitfly, worm, and fission yeast (Figure 3A; Figure S3). Transcriptional activation of the Arabidopsis *CTF18* gene in *E2Fa-DPa*-overexpressing plants was confirmed by quantitative real-time PCR analysis (Figure 3B). The spatial expression pattern of *CTF18* was analyzed in more than six independent transgenic lines expressing the β-glucuronidase (*GUS*) reporter gene under control of the *CTF18* promoter. In 7-day-old seedlings, the levels of *CTF18* expression were high at the shoot apical and root meristems (Figure 3C and 3D), corresponding with an anticipated role for CTF18 during cell cycle progression.

To investigate the role of the plant CTF18 in the establishment of sister chromatid cohesion, we investigated the loss-of-function effect of CTF18 in T-DNA insertion mutant nuclei. In plants with the T-DNA inserted into the 7th intron (*ctf18-1*; ecotype Nossen-0 [Nos-0]), the *CTF18* transcript level was reduced by 85% compared to that in control plants, whereas in plants with the T-DNA inserted into the 19th exon (*ctf18-2*; ecotype Columbia-0 [Col-0]) (Figure 3E), no transcripts were detectable (Figure 3F). FISH analysis with the BAC clones T2P11 and T7N9 on *ctf18-2* leaf nuclei revealed sister chromatid separation in 40.5% of homologous chromosomes versus 28.1% in the wild type (P-value <0.001; Figure 3G and 3H), demonstrating that CTF18 contributes to sister chromatid cohesion. These observations reveal that the function of the CTF18 protein is highly conserved in eukaryotes. Again, as observed for the *etg1-1* mutants, the frequency of centromeric signals did not change significantly (Figure 3I).

### Sister chromatid cohesion is crucial for development of *ETG1*-deficient plants

**Figure 4:**
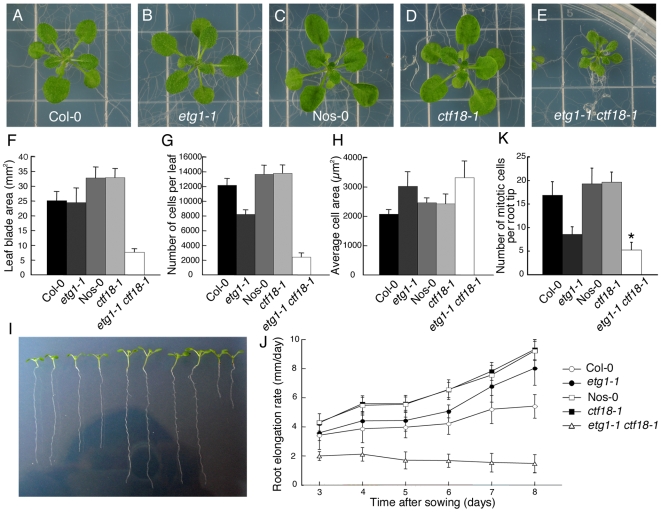
Inhibition of plant growth by loss of sister chromatid cohesion. (A–E) Seedling phenotypes of 21-day-old wild-type (Col-0) (A), *etg1-1* (*B*), wild-type (Nos-0) (C), *ctf18-1* (D), and *etg1-1 ctf18-1* (E) grown on MS plates. (F-H) Leaf growth of the first leaf pair of wild-type (Col-0), *etg1-1*, wild-type (Nos-0), *ctf18-1*, and *etg1-1 ctf18-1* plants. Leaf blade area (F), epidermal cell number (G), and average epidermal cell size (H) on the abaxial side of the leaf. Data represent average ± SD (n = 5). (I) Root phenotype of 8-day-old wild-type (Col-0), *etg1-1*, wild-type (Nos-0), *ctf18-1*, and *etg1-1 ctf18-1* plants grown on MS plates (two plants each). (J) Kinematic root growth analysis of the root elongation rate of wild-type (Col-0), *etg1-1*, wild-type (Nos-0), *ctf18-1*, and *etg1-1 ctf18-1* plants. Plants were grown on MS agar plates. Data represent average ± SD (n = 20). (K) Number of mitotic cells per root tip of 7-day-old wild-type (Col-0), *etg1-1*, wild-type (Nos-0), *ctf18-1*, and *etg1-1 ctf18-1* seedlings. Data represent average ± SD (n = 20 to 30). Asterisk marks statistically significant differences by Student's *t*-test (P-value <0.05).

To analyze the genetic interaction between *etg1* and other sister chromatid cohesion mutants, double mutants were constructed for *etg1* and *ctf18*. Single mutants were viable and developed normally (Figure 4A–4D). By contrast, *etg1-1 ctf18-1* double mutants had distinctly smaller leaves (Figure 4E), a phenotype observed for *etg1-2 ctf18-1* and *etg1-2 ctf18-2* as well (Figure S4). When the first leaf pair from each mature plant was compared, the leaf blade area of *etg1-1 ctf18-1* double mutants was strongly reduced, whereas those of the *etg1-1* and *ctf18-1* single mutants was nearly identical to those of the control lines (Figure 4F). Furthermore, the total abaxial pavement cell number per leaf was considerably lower in *etg1-1 ctf18-1* mutants than that in control plants (Figure 4G), whereas their cell area was similar to that of the *etg1-1* single mutant (Figure 4H), indicating a stringent cell division arrest in the double mutant. The growth defect was also observed in *etg1-1 ctf18-1* roots, with a significantly reduced root growth rate (Figure 4I and 4J) and a significantly lower number of dividing cells in the root meristem (metaphase, anaphase, and telophase) in the *etg1-1 ctf18-1* mutant than observed in the single mutant and wild-type plants (Figure 4K).

**Figure 5:**
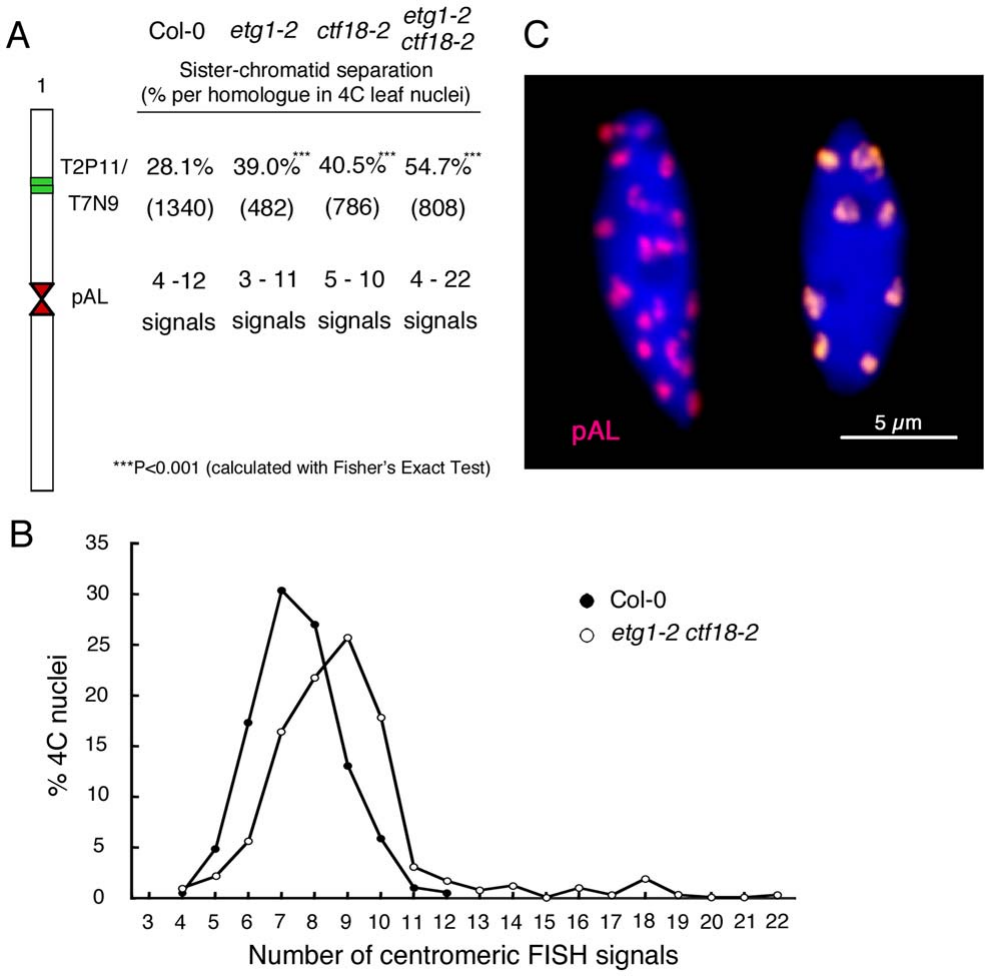
Synergistic effect of *etg1* and *ctf18* on sister chromatid cohesion in 4C leaf nuclei. (A) Percentage of positional separation frequencies (number of investigated nuclei in parentheses) analyzed in wild-type (Col-0) and *etg1-2 ctf18-2* mutant plants after FISH with the labeled T2P11 BAC from chromosome 1. The FISH probe pAL detected the centromeric 178-bp repeats. More than the expected 20 signals from separated sister centromeres in 4C nuclei might be caused by signal splitting due to pAL repeat elongation along the centromeres. (B) Frequency of centromeric signals in the *etg1 ctf18* double mutant compared to wild-type nuclei. (C) Sister centromere separation in an *etg1 ctf18* nucleus (left) compared to aligned sister centromeres in a wild-type nucleus (right). Due to occasional centromere fusion, the average signal number in the wild-type is 8 instead of 10.

FISH analysis revealed that the cohesion phenotype observed in the *etg1-2* and *ctf18-2* single mutants was aggravated in the double mutant, displaying sister arm separation in up to 54.7% of homologous chromosomes (Figure 5A). Moreover, in contrast to the single mutants, centromere cohesion was clearly impaired in the double mutant, as indicated by the increase in number of centromeric signals per nucleus in *etg1-2 ctf18-2*. Up to 22 signals could be observed (compared to up to maximum 12 signals in wild-type nuclei), indicating a strong defect in centromere cohesion (Figure 5A–5C).

*Arabidopsis* cohesins contain four alternative SCC1 homologs (SYN1 to SYN4) with different functions during somatic and meiotic cell cycles (reviewed in [31]). The impact of *etg1* on plant growth and sister chromatid cohesion was confirmed by crosses between *etg1* and the *syn4* cohesin mutant, also known as *rad21.3* [32]. SYN4 represents an *Arabidopsis* α-kleisin protein required for cohesion along chromosome arms and at centromeres [33]. As observed for the *etg1 ctf18* plants, the *etg1 syn4* double mutant plants had smaller leaves than the control plants, correlating with a significantly reduced cell number (Figure S5). The synergistic effects of the *etg1* and the cohesion mutants *ctf18* and *syn4* on cell division strengthen the hypothesis that ETG1 is required for sister chromatid cohesion and illustrate the importance of sister chromatid cohesion for correct development of plants lacking ETG1.

### Sister chromatid cohesion is required for DNA repair of *etg1* mutants

**Figure 6:**
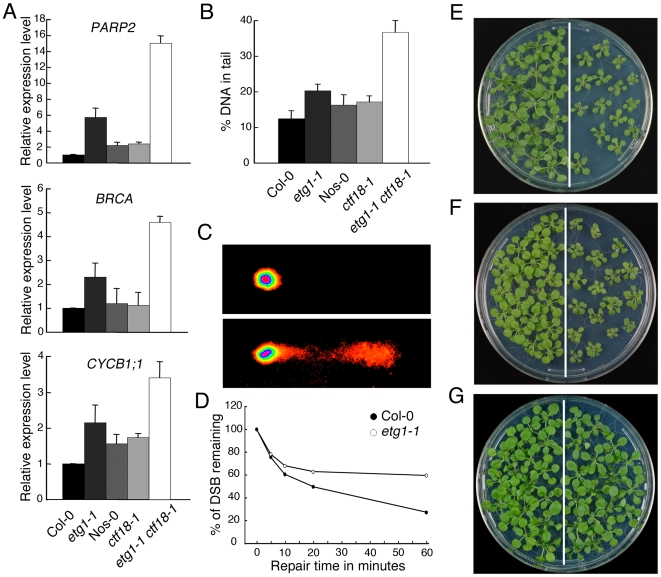
Inefficient DNA repair upon loss of sister chromatid cohesion. (A) Real-time RT-PCR analysis of DNA stress-inducible genes *PARP2*, *BRCA*, and *CYCB1;1* in wild-type (Col-0), *etg1-1*, wild-type (Nos-0), *ctf18-1*, and *etg1-1 ctf18-1* plants. Total RNA prepared from 8-day-old seedlings was amplified by RT-PCR. All values were normalized against the expression level of the *ACTIN2* gene. (B) Statistical analysis of a comet assay. The average %-values of DNA in tails of nuclei of 7-day-old wild-type (Col-0), *etg1-1*, wild-type (Nos-0), *ctf18-1*, and *etg1-1 ctf18-1* seedlings. Error bars indicate SD. (C) Examples of comets from plant nuclei with undamaged (top) or damaged (bottom) DNA. (D) Kinetics of DSB repair in wild-type versus *etg1-1* mutant plants. Fractions of remaining DSB were calculated for 0, 5, 10, 20, and 60 min recovery time after treatment with 50 µg/ml bleomycin for 1 h. Maximum damage was normalized as 100% at t = 0. (E–G) Wild-type (Col-0, left) and *etg1-1* (right) plants were grown on medium holding 50 ppm methyl methane sulfonate (E), 3 µg/ml mitomycin C (F), or no supplement (G). Plants were photographed 3 weeks after sowing.

Previously, we reported that ETG1*-*deficient plants suffer from replication stress, resulting in transcriptional induction of DNA repair genes and activation of the DNA replication checkpoint [24]. To assess the level of DNA damage in cohesion mutants, we compared the expression levels of the marker genes coding for poly(ADP-ribose) polymerase 2 (*PARP2*), breast cancer (*BRCA*), and B-type cyclin 1 (*CYCB1;1*) by real-time reverse-transcription (RT)-PCR in wild-type versus *etg1-1*, *ctf18-1*, and *etg1-1 ctf18-1* mutant plants. γ-irradiation, UV light, and radiomimetic agents (such as bleomycin) are known to induce *PARP2*, *BRCA*, and *CYCB1;1* expression [29],[34]. The expression level of these DNA stress genes was significantly upregulated in the *etg1-1* mutant, confirming previous data [24]. By contrast, the cohesion mutant *ctf18-1* showed no signs of DNA stress (Figure 6A). Interestingly, expression of the *PARP2*, *BRCA*, and *CYCB1;1* genes was hyper-induced in *etg1-1 ctf18-1* double mutant plants (Figure 6A), indicating severe DNA stress. To examine the level of DNA damage, 8-day-old seedlings were assayed by comet assay. In agreement with previous analyses, *etg1-1*, but not *ctf18-1*, mutants, exhibited significant DNA damage in comparison with control plants, whereas the DNA damage level of the *etg1-1 ctf18-1* double mutant seedlings was much higher than that observed in the single *etg1-1* mutants (Figure 6B and 6C).

In *Arabidopsis,* the S phase-established cohesion is seemingly a prerequisite for double-strand break (DSB)-dependently enforced cohesion that, in turn, is required for homologous recombination repair between sister chromatids [35]. Therefore, the DSB repair, kinetics of *etg1-1* and wild-type plants were compared during the recovery from bleomycin treatment by calculating the extent of the remaining DNA damage from the percentage of DNA in the comet tails. Whereas in wild-type seedlings almost 80% of all DSBs was repaired within 1 h, in *etg1-1* plants, it was significantly delayed (Figure 6D). Moreover, the *etg1-1* mutants displayed increased sensitivity to methyl methane sulfonate (a monofunctional alkylating agent) and mitomycin C (a multifunctional DNA cross-linking agent), both triggering DSBs indirectly by interfering of DNA excision repair with DNA replication (Figures 6E–6G). In replicating cells, such DSBs are preferentially repaired through homologous recombination that needs an intact sister chromatid in physical proximity. These data substantiate a role for ETG1 in sister chromatid cohesion and support the idea that cohesion might be important for homologous recombination repair.

### Knockdown of the human ETG1 results in defective chromatid cohesion

**Figure 7:**
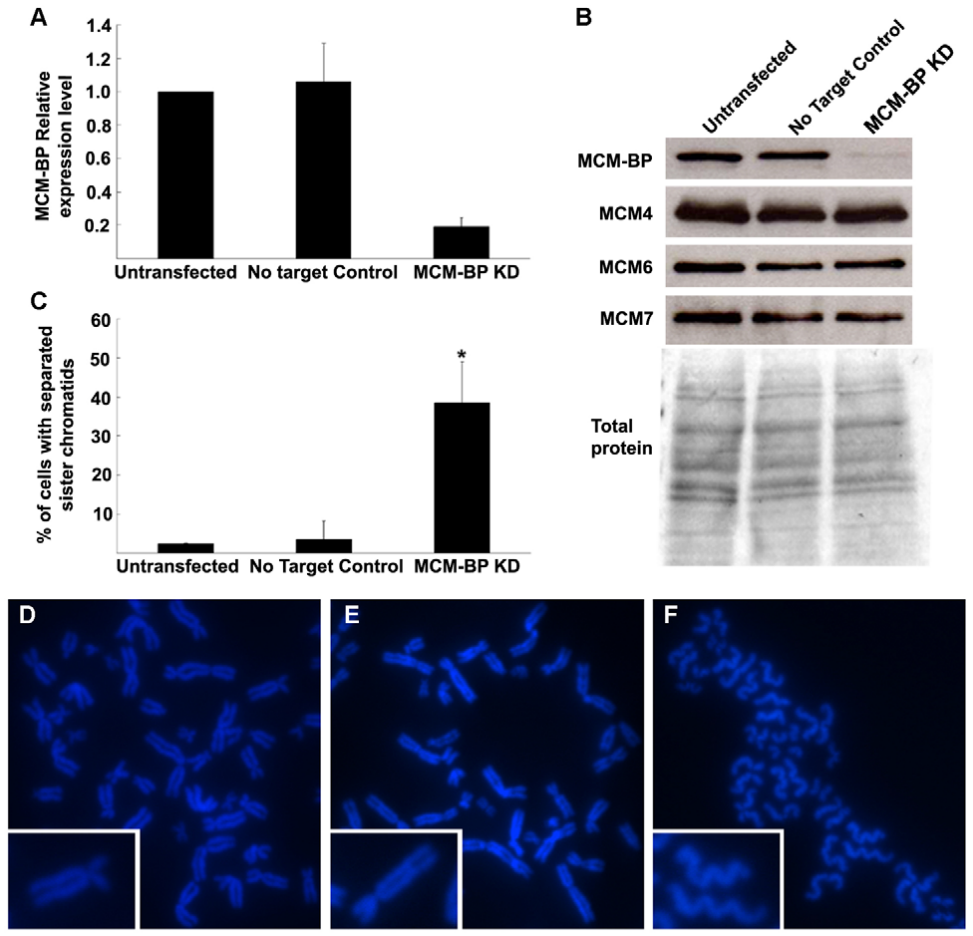
Induction of cohesion defects by downregulation of human *MCM-BP*. (A) *MCM-BP* transcript levels in siRNA transfected HEK-293T cells versus untransfected and transfected control cells. Samples were harvested 48 h after transfection. All values were normalized against the expression level of the *TBP* and *UBC* genes. (B) MCM-BP, MCM4, MCM6, and MCM7 protein levels in samples as described in (A). (C) Quantification of nuclei showing totally separated sister chromatids (n>100 per condition, obtained from two independent transfection experiments). The asterisk marks a significant difference by Student's *t*-test (P-value <0.05). (C–E) Representative images of mitotic spread of untransformed (D), control transfected (E), and *MCM-BP* siRNA-transfected HEK-293T cells (F). Insets show higher magnification images of single sister chromatid pairs.

A role for MCM-BP, the human ETG1 homolog, in sister chromatid cohesion was assessed with the RNA interference technique by means of pooled short interference RNAs (siRNAs). In human embryonic kidney 293T (HEK-293T) cells transfected with siRNAs that targeted MCM-BP, the *MCM-BP* expression was much lower than that of untransfected or transfected control cells (Figure 7A). Protein gel blotting with a specific antibody confirmed that the decrease in *MCM-BP* transcription was accompanied by a reduced MCM-BP protein abundance (Figure 7B). No change in protein level was seen for MCM4, MCM6, and MCM7, which are the most conserved and core subunits of the MCM complex, demonstrating that the siRNAs targeted specifically MCM-BP without affecting the abundance of other proteins of the MCM complex. As seen in mitotic spreads (Figure 7C–7F), depletion of MCM-BP strongly affected sister chromatid cohesion, resulting in a higher proportion of nuclei with completely separated sister chromatids (38% versus 2% or 3% in untransfected or transfected control cells, respectively). Thus, as observed for its plant counterpart, MCM-BP seems to be important for sister chromatid cohesion.

## Discussion

### ETG1 is a replisome component involved in establishment of sister chromatid cohesion

Previously, we have demonstrated that the ETG1 protein binds to replisome components, is required for efficient DNA replication, and its absence causes DNA replication stress [24]. Here, we showed that ETG1 plays an additional role in sister chromatid cohesion. The effects of *ETG1* deficiency on cohesion might result from impaired DNA replication, rather than vice versa, because no DNA stress was observed in *ctf18* mutants displaying a loss in sister chromatid cohesion similar to that of *etg1* knockout plants. Cohesion was lost at mid-arm positions, but not at the centromeres. Only when the *etg1* mutation was introgressed into the *ctf18* mutant background, additional loss of sister centromere cohesion was observed. Cohesin binding at centromeric DNA is particularly important because centromeres are directly exposed to spindle pulling forces during mitosis and must resist sister chromatid segregation until all chromosomes are bipolarly attached to the spindle. Enhanced cohesion at centromeres compared to chromosome arms has been observed in yeasts and mammals [5]. In wild-type nuclei of *Arabidopsis*, cohesion along the arms is less consistent than at the centromeres, suggesting that also in plants centromere cohesion is enforced [36],[37]. The preferential release of sister arm cohesion after *ETG1* depletion might be explained by a lower cohesin concentration at the arms than at the centromeres. Alternatively, additional proteins that are not affected by the loss of *ETG1* might be needed for centromere cohesion. In yeasts and mammals, the shugoshin (SGO) protein is essential for protection of centromere cohesion [38]. The recruitment of SGO to centromeres in these species depends on the checkpoint protein BUB1 and on HP1α [39]–[41]. Interestingly, the putative *Arabidopsis* ortholog of BUB1 is transcriptionally upregulated in *ETG1*-deficient plants. This finding suggests that the cohesion defect in *etg1* activates the spindle checkpoint to prevent centromeres from segregating.

Several observations suggest that the cohesion establishment is coupled to the replication process and occurs in close vicinity to the replisome. Cohesion is initiated by the acetyltransferase ECO1/Ctf7 that travels along the DNA together with replication forks [16]. Furthermore, in yeasts, the depletion or mutation of several nonessential components of the replisome, such as the replication factor Ctf18, causes cohesion defects [19]–[21]. Knockout mutation of the *Arabidopsis* CTF18 homolog impairs sister chromatid cohesion as well. Through its association with MCM proteins, ETG1 is very likely a component of the replisome and plays a role in establishing cohesion during the replication process rather than in cohesin loading. This model is supported by co-regulated expression of *ETG1* and other genes involved in cohesion establishment, such as *ECO1*/*CTF7*, *CHL1*, and *CTF18* that are expressed during S phase and, interestingly, are transcriptionally upregulated in *E2Fa-DPa*-overexpressing plants [30]. A role in cohesion establishment is also suggested by the synergetic effect between *etg1* and *ctf18* on sister chromatid cohesion. Additionally, the human MCM-BP was found to associate preferentially with chromatin during G1/S and S, corresponding with the timing of cohesion establishment, whereas cohesion loading occurred immediately after formation of the nuclear envelope in telophase [25],[42]. Taken together, these results imply that ETG1 functions during S phase for cohesion establishment and that it represents a novel important link between DNA replication and sister chromatid cohesion. As ETG1 is part of the MCM complex, it is not unlikely that the MCM subunits contribute to cohesion establishment as well, however, but until now, no such role has been reported for MCMs in yeasts and mammals.

### Impaired sister chromatid cohesion enhances pre-mitotic arrest after DNA damage

Cohesion is essential after replication to allow homologous recombination repair of DNA DSBs that might have arisen by genotoxic impact directly or indirectly by interference of excision repair with replication-mediated gaps. In fission yeast (*Schizosaccharomyces pombe*), the cohesion component SCC1/RAD21 was first identified in genetic screens for mutants that are hypersensitive to DNA damage [43]. Also SYN2/RAD21.1-deficient plants are hypersensitive to ionizing radiation [32] and SMC5/6 complex proteins, as well as the S phase-dependent cohesin component SYN1, are needed to enhance sister chromatid alignment required for homologous recombination after induction of DSBs by X-irradiation [35]. Although CTF18 is necessary for sister chromatid cohesion, the corresponding knockout plants are viable and develop normally, without any significant effect on cell cycle progression. These data indicate that mild chromosome cohesion defects (up to 40% of sister chromatid separation) are not that critical for cell cycle progression and plant development. However, the more pronounced cohesion deficiency in the *etg1 ctf18* double mutant has a strong impact on plant growth: leaf size is severely reduced and root growth is inhibited as a consequence of restricted cell division. This phenotype correlates with an increased loss of sister chromatid cohesion and a strong increase in the expression of DNA damage response genes. While in the *etg1* single mutants the developmental disturbance caused by endogenous DNA stress is weak, in the *etg1 ctf18* double mutant, the synergetic effect on development indicates that cohesion of the sister chromatids is highly important for correct DNA repair. We assume that the *etg1 ctf18* double mutants are less efficient in correct DNA repair by homologous recombination because of the reduced sister arm cohesion, reminiscent of the *smc5/6* mutants that are hypersensitive to genotoxins and defective in homologous recombination [35]. This model is supported by slower DSB repair kinetics in *ETG1*-deficient seedlings than in wild-type plants and by their increased sensitivity to genotoxins. The presumed disturbance of homologous recombination repair in *etg1 ctf18* plants might enforce a more stringent checkpoint than that in the single *etg1* mutants, thus inhibiting growth by strongly reduced cell division. These results underscore the importance of cohesion for the development of DNA damage-suffering plants. In mammals and yeasts, the spindle checkpoint arrests cells in mitosis upon incorrect mitotic spindle attachment to the chromosomes [44]. Depletion of the origin recognition complex (Orc2) protein of budding yeast delays progression through mitosis because of impaired sister chromatid cohesion and activation of both the DNA damage and the Mad2 spindle checkpoints [45]. Whether the induction of spindle checkpoint or rather of DNA damage checkpoint in the single *etg1* and in *etg1 ctf18* double mutants contributes to the observed G2/M arrest remains to be tested.

Except for mediating DNA repair, cohesion is essential for diverse biological processes, including chromosome segregation and gene expression [46]. The importance of cohesion is illustrated by the observations that defects in cohesion factors associate with human genetic disorders, including colorectal cancer and developmental diseases, such as the Cornelia de Lange syndrome [47],[48]. Studies from a number of organisms have shown that defects in sister chromatid cohesion lead to chromosome mis-segregation, with aneuploidy, a hallmark of cancer progression, as a consequence [48],[49]. Similarly to its plant counterpart, the depletion of the human MCM-BP protein caused chromatid cohesion defects, suggesting that aberrant *MCM-BP* expression might result in chromosome instability that increases the organism's risk of neoplastic transformation.

## Materials and Methods

### Plant growth conditions and plasmid construction

*Arabidopsis thaliana* (L.) Heyhn. (ecotypes Columbia-0 [Col-0] and Nossen-0 [Nos-0]) plants were grown under long-day conditions (16 h/8 h light/darkness) at 22°C on half-strength Murashige and Skoog (MS) agar plates [50]. The *ctf18-1* (13-0845-1) and *ctf18-2* (SALK_126071) alleles were retrieved from the Salk Institute Genomic Analysis Laboratory engine (http://signal.salk.edu/cgi-bin/tdnaexpress) and the seeds were acquired from the RIKEN BioResource Centre and Arabidopsis Biological Research Center, respectively. To screen for homozygous insertion alleles, the following primer pairs were designed: 5′-TCACATTGCAGCTAAGCATTG-3′ and 5′-GCTAACGTGTACCGGAGACAG-3′ for *ctf18-1*, and 5′-ACAACTGGCGGGTTTGGTCATG-3′ and 5′-TTCTAACGGGTCTCTTCACAGC-3′ for *ctf18-2*. The *CTF18* promoter sequence was amplified from *Arabidopsis* genomic DNA by PCR with the 5′-GGGGACAAGTTTGTACAAAAAAGCAGGCTTATGAGTTCATAGCTGACTCATCC-3′ and 5′-GGGGACCACTTTGTACAAGAAAGCTGGGTCCTCCTCCGGCAATGGGATATCG-3′ primers. The PCR fragment was cloned into the pDONR201 entry vector by BP recombination reaction and subsequently transferred into the pKGWFS7 destination vector [51] by LR recombination reaction, resulting in a transcriptional fusion between the *CTF18* promoter and the enhanced green fluorescent protein and GUS (*eGFP::GUS*) gene. The construct was transferred into the *Agrobacterium tumefaciens* C58C1Rif^R^ strain harboring plasmid pMP90. The obtained *Agrobacterium* strains were used to generate stably transformed *Arabidopsis* with the floral dip transformation method [52]. Transgenic plants were grown on kanamycin-containing medium and later transferred to soil. The *etg1-1*, *etg1-2*, and *atrad21.3/syn4* mutants and the *E2Fa/DPa*-overexpressing plants have been described previously [24],[32],[53].

### Phenotypic analysis

Plants were germinated and grown in round 12-cm Petri dishes filled with 100 ml of half-strength MS medium (Duchefa, Haarlem, The Netherlands) and 0.8% plant tissue culture agar (Lab M, Bury, UK). Three-week-old plants were harvested, cleared overnight in 100% ethanol, and subsequently stored in lactic acid for microscopy. The leaf primordia were observed under a microscope fitted with differential interference contrast optics (DMLB; Leica, Wetzlar, Germany). The total (blade) area of the first leaves of each seedling was determined from drawing-tube images with the public domain image analysis program ImageJ (version 1.30v; http://rsb.info.nih.gov/ij/). The primordia were digitized directly with a charge-coupled device camera mounted on a binocular (Stemi SV11; Zeiss, Jena, Germany), connected to a personal computer fitted with a frame-grabber board LG3 (Scion Corp., Frederick, MD, US2). Cell density was determined from scanned drawing-tube images of outlines of at least 30 cells of the abaxial epidermis located 25% and 75% from the distance between the tip and the base of the leaf primordium, halfway between the midrib and the leaf margin. The following parameters were determined: total area of all cells in the drawing, total number of cells, and number of guard cells. From these data, the average cell area was calculated and the total number of cells per leaf estimated by dividing the leaf area by the average cell area (averaged between the apical and basal positions).

### Quantitative PCR analysis

RNA was extracted from *Arabidopsis* tissues with RNeasy Plant Mini Kit (Qiagen, Hilden, Germany). First-stranded cDNA was prepared from total RNA with the Superscript III First-Strand Synthesis System (Invitrogen, Carlsbad, CA, USA) according to the manufacturer's instructions. For quantitative PCR, a LightCycler 480 SYBR Green I Master (Roche Diagnostics, Brussels, Belgium) was used with 100 nM primers and 0.1 µg of RT reaction product. Reactions were run and analyzed on the LightCycler 480 Real-Time PCR System (Roche Diagnostics) according to the manufacturer's instructions. Quantitative reactions were done in triplicate and averaged. Primers used were 5′-GGCTCCTCTTAACCCAAAGGC-3′ and 5′-CACACCATCACCAGAATCCAGC-3′ for *ACTIN2*, 5′-TAATGACGCTTCTGGCAGTG-3′ and 5′-CATGGTAGTGGAGCTGCAAA-3′ for *CTF18*, 5′-ATGGCGTTCTGCTCCTCTGC-3′ and 5′-GGTGCTGTTTTCCCCACACC-3′ for *PARP2*, 5′-TGTTCCCTCTTTCAGCGATTTGATG-3′ and 5′-GGCCTCTGAGTCCATTCAAACA-3′ for *BRCA*, 5′-CTCAAAATCCCACGCTTCTTGTGG-3′ and 5′-CACGTCTACTACCTTTGGTTTCCC-3′ for *CYCB1;1*, 5′-GCTAGCTCCATGGGACAGAG-3′ and 5′-CCCCAAACTCCAAATGTCAC-3′ for *NQK1*, 5′- TAGGAGCAGCAATGCATCAG-3′ and 5′-CTGCAATGTCAAGCCCTCTT-3′ for *PLEIADE*, 5′-TCTGCGGCTCTACGGTTACT-3′ and 5′-CTCTAGCCAATGACGCAACA-3′ for *AURORA2*, 5′-GTGGCAAGCCTTCTTCACTC-3′ and 5′-TCCTTTTCCCTGACATTTGC-3′ for *MYB3R4*, 5′-TTGATTGCTAATCCACAGATGG-3′ and 5′-AAGCGTGTCGACTTTGTGAA-3′ for *MAD2*, and 5′- CCTAGGATCTCATCATTACTCTACACC-3′ and 5′- CCATGTATCCTCGTACGGAGTTCC -3′ for *CDKA;1*.

### Histochemical GUS measurements

Histochemical GUS assays were carried out according to standard protocols [54]. The young seedlings were incubated in 80% acetone for 2 h at 4°C. After the material had been washed in phosphate buffer, it was immersed in the enzymatic reaction mixture (1 mg/mL of 5-bromo-4-chloro-3-indolyl β-d-glucuronide, 2 mM ferricyanide, and 0.5 mM of ferrocyanide in 100 mM phosphate buffer, pH 7.4). The reaction was carried out overnight at 37°C in the dark. The material was cleared with chlorolactophenol (chloral hydrate/phenol/lactic acid 2∶1∶1) and observed under a light microscope or a stereoscope.

### Root growth analysis

For root growth experiments, seedlings were grown in square plates in vertical position in half-strength MS medium containing 10 g/L plant tissue culture agar. Root growth was marked every 24 h on plates that were photographed and was measured with ImageJ software by calculating the distance between successive marks along the root axis.

### Determination of the mitotic index

Roots were fixed in a solution of formaldehyde, ethanol, and acetic acid (2∶17∶1) for 12 h at 4°C, washed twice in water, and mounted under cover slips. The samples were crushed, snap-frozen with liquid nitrogen to remove the cover slip, and mounted in Vectashield (Vector Laboratories, Burlingame, CA, USA) containing 1 µg/ml 4′,6-diamidino-2-phenylindole (DAPI). The roots were analyzed for mitotic stages with an Axiovert fluorescence microscope (Zeiss).

### Microarray and GO analysis

For the microarray experiment, RNA was extracted from 9-day-old *Arabidopsis* leaf primordia with the RNeasy Plant Mini Kit (Qiagen). The microarray experiment was done by the VIB MicroArrays Facility (Leuven, Belgium; http://www.microarrays.be/) with the ATH1 GeneChip array (Affymetrix, Santa Clara, CA, USA) of 23,800 probe sets designed for *Arabidopsis*. The experimental design comprised three replicates of each genotype, with one replicate corresponding to one RNA extraction from an independent pool of plants. Raw data obtained by microarray were analyzed as described [55]. To determine significantly overrepresented GO categories among up- and down-regulated genes, we used the BiNGO plugin for Cytoscape (http://www.psb.ugent.be/cbd/papers/BiNGO/) [27]. Promoter motif enrichment was calculated with the hypergeometric distribution based on MSA motif instances as reported [56].

### FISH analysis, microscopic evaluation, image processing, and statistics

Preparation of nuclei, probe labeling, and fluorescent in situ hybridization (FISH) were as described [57]. FISH signals were analyzed with an epifluorescence microscope Axiophot (Zeiss) with a 100x/1.45 α-plan-fluar objective and a 3-chip color camera (DXC-950P; Sony, Tokyo, Japan). The microscope was integrated into a Digital Optical 3D Microscope system (Schwertner GbR, Jena, Germany) to check signal separation/distances along x-, y-, and z-axes. Images were captured separately for each fluorochrome with appropriate excitation and emission filters. The images were merged with Adobe Photoshop 6.0 software (Adobe Systems, San Jose, CA, USA). FISH signals indicating positional sister chromatid separation were compared against those of the Col-0 wild-type by the one-sided Fisher's exact test.

### Comet assay for DNA damage measurement

DNA damage was detected by comet using a CometAssay kit (Trevigen, Gaithersburg, MD, USA). Samples were prepared as described [58]. The percentage of DNA in each comet tail was evaluated with Comet Score software (http://www.autocomet.com). DNA damage was calculated by averaging the values for the percentage of DNA in tails from three individual slides, scoring 80 comets per slide. The percentage of the remaining damage after a given post-treatment recovery time is defined as: % of DSB remaining  =  (mean % tail-DNA (tx) - mean % tail-DNA (control)) / (mean % tail-DNA (t0) - mean % tail-DNA (control))×100.

### HEK-293T cell culture and transfection

HEK-293T cell cultures were grown in 5 ml of complete medium (Dulbecco's modified Eagle medium with 10% fetal calf serum; Invitrogen) at 37°C and 5% CO_2_. siRNAs were transfected into HEK-293T cells grown in 6-well plates according to the manufacturer's instructions (DharmaFECT, Thermo Fisher Scientific, Waltham, MA, USA). Final concentrations of each siRNA were 30 nM. The following siRNA sequences were used: human MCM-BP (C10ORF119) (SMARTpool; J-014474-09, J-014474-10, J-014474-11, and J-014474-12) and control (SMARTpool non-targeting pool).

### Protein gel blotting

Protein extracts were prepared from 2-day-old transfected HEK293T cells. Protein gel blotting was carried out according to standard procedures with primary anti-MCM4 (ab4459), anti-MCM6 (ab4458), and anti-MCF7 (ab52489) antibodies (Abcam, Cambridge, UK) at a dilution of 1∶2,000, 1∶2,000, and 1∶10,000, respectively. The MCM-BP antibody [25] was used at a 1∶1,000 dilution and a horseradish peroxidase–conjugated donkey anti-rabbit (GE-Healthcare) diluted 1∶10,000 as a secondary antibody. Proteins were detected with Western Lightning Plus-ECL luminol reagent (Perkin Elmer, Massachusetts, USA) according to the manufacturer's instructions.

### Chromosome spreads and DAPI staining

Sub-confluent HEK cells were treated for 48 h after transfection with KaryoMAX colcemid (Invitrogen) to enrich for mitotic chromosomes. The complete medium was replaced by 2 ml of medium at a final concentration of KaryoMAX of 0.6 µg/ml. Cells were incubated at 37°C with 5% CO_2_ for 5 h before harvesting, trypsinized, pelleted (110 g for 5 min), and resuspended in 1 ml of a hypotonic solution of KCl at a final concentration of 60 mM for 30 min at room temperature. After incubation, HEK cells were twice pelleted (110 g for 5 min) and resuspended in freshly made methanol:glacial acetic acid (3∶1) added drop-wise. Two or 3 drops of suspended cells were applied to precleaned smear glass slides (Menzel-Gläzer, Braunschweig, Germany) and chromosomes were counterstained with VectaShield (Vector Laboratories, Burlingame, CA, USA) containing DAPI. A minimum of 200 mitotic spreads were imaged for each control or siRNA-treated cell population with the DAPI channel of a BX61 Olympus epifluorescence microscope equipped with a 100×/1.30 UPlan FLN objective coupled to a U-C MAD 3 imaging system with a Cell∧M imaging software (Olympus, Tokyo, Japan).

### Quantitative PCR analysis of MCM-BP knocked-down cells

HEK-293T cells were collected 48 h after transfection with a rubber policeman. RNA was extracted with an RNeasy animal Mini Kit (Qiagen) and cDNA was prepared with the cDNA Synthesis System according to manufacturer's instructions (Roche Diagnostics, Indianapolis, USA). For quantitative PCR, a LightCycler 480 SYBR Green I Master (Roche Diagnostics) was used with 100 nM primers and 0.1 µg of reverse transcription reaction product. Reactions were run and analyzed on the LightCycler 480 RealTime PCR System according to manufacturer's instructions (Roche Diagnostics). All quantifications were normalized to the *TATA Binding Protein* (*TBP*) and *Ubiquitin C* (*UBC*) expression levels. Quantitative reactions were done in triplicate and averaged. Primers used were 5′ACTCTCCACGAAATACCACTTTG3′ and 5′GTAGGATGTTGAGGGACTGACTCG3′ for *MCM-BP,* 5′CGGCTGTTTAACTTCGCTTC3′ and 5′CACACGCCAAGAAACAGTGA3′ for *TBP*, and 5′ATTTGGGTCGCGGTTCTTG3′ and 5′TGCCTTGACATTCTCGATGGT3′ for *UBC*.

## Supporting Information

Figure S1 Upregulation of mitotis-specific genes in *etg1* mutants. Real-time RT-PCR analysis of mitosis-specific genes *PLEIADE (PLE)*, *KNOLLE (KN)* , *AURORA 2 (AUR2)* , *MYB3R4*, and *CDKA;1* (as a control) in wild-type (Col-0; white bars) and *etg1-1* (black bars) plants. Total RNA prepared from the first leaf of 8-day-old seedlings was amplified by RT-PCR. All values were normalized against the expression level of the *ACTIN2* gene.
(0.11 MB TIF)

Figure S2 Upregulation of cohesion establishment genes in E2Fa-DPa-overexpressing plants. Relative expression level of cohesion establishment genes *ECO1, CHL1*, and *CTF18* in wild-type (black) and *E2Fa-DPa* -overexpressing (white) plants. Data were imported from [25].
(0.07 MB TIF)

Figure S3 Conservation of the CTF18 protein in eukaryotes. Alignment of Arabidopsis CTF18 (ATCTF18) and its orthologous proteins: Os03g0264800 (rice), CHTF18 (NP_071375; human), Chtf18 (NP_663384; mouse), cutlet (NP_787969; fruitfly), K08F4.1 (NP_501841; C. elegans), CTF18 (NP_013795; budding yeast), and chl12 (NP_595200; fission yeast). Amino acid similarity between ATCTF18 and its orthologous proteins is 45% for rice, 30% for human, 30% for mouse, 29% for fruitfly, 27% for *C. elegans*, 26% for budding yeast, and 24% for fission yeast.
(3.44 MB TIF)

Figure S4 Genetic interaction between *etg1* and *ctf18* mutants on plant growth. (A-D) Seedling phenotypes of 21-day-old wild-type (Col-0) (A), etg1-2 (Col-0 background) (B), ctf18-2 (Col-0 background) (C), and *etg1-2 ctf18-2* (D) plants. (E-I) Seedlings phenotype of 21-day-old wild-type (Col-0) (E), *etg1-2* (Col-0 background) (F), wild-type (Nos-0) (G), *ctf18-1* (Nos-0 background) (H), and *etg1-2 ctf18-1* (I) plants.
(2.33 MB TIF)

Figure S5 Genetic interaction between *etg1* and cohesin mutant *syn4*. (A-D) Seedling phenotypes of 21-day-old wild-type (Col-0) (A), *etg1-2* (Col-0 background) (B), *syn4* (Col-0 background) (C), and *etg1-2 syn4* (D) plants. (E-G) Leaf growth of the first leaf pair of 21-day-old wild-type (Col-0), *etg1-1, syn4*, and *etg1-1 syn4* plants. Leaf blade area (E), epidermal cell size on the abaxial side of the leaf (F), and epidermal cell number on the abaxial side of the leaf (G). Data represent average ± SD (n = 5).
(1.86 MB TIF)

Table S1 Upregulated genes in *etg1* compared with the wild-type genes (Col-0).
(0.20 MB DOC)

Table S2 Downregulated genes in *etg1* compared with the wild-type genes (Col-0).
(0.06 MB DOC)
