# Supplementary figures and images for: The MCM-Binding Protein ETG1 Aids Sister Chromatid Cohesion Required for Postreplicative Homologous Recombination Repair
Source: PLoS Genet. 2010 Jan 15;6(1):e1000817. doi: 10.1371/journal.pgen.1000817 (PMC2806904; doi:10.1371/journal.pgen.1000817)

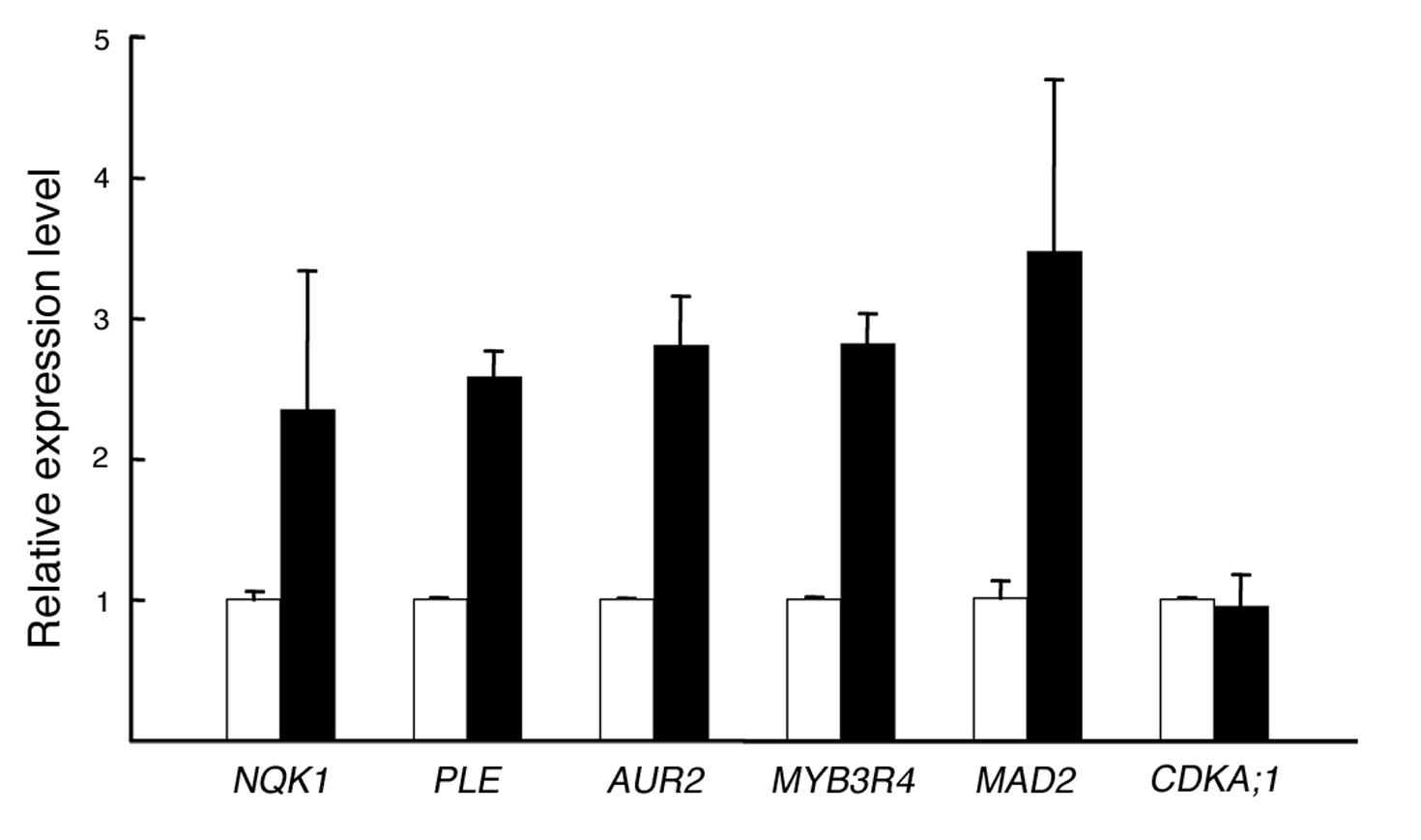

Supplement: Figure S1 — Upregulation of mitotis-specific genes in etg1 mutants. Real-time RT-PCR analysis of mitosis-specific genes PLEIADE (PLE), KNOLLE (KN) , AURORA 2 (AUR2) , MYB3R4, and CDKA;1 (as a control) in wild-type (Col-0; white bars) and etg1-1 (black bars) plants. Total RNA prepared from the first leaf of 8-day-old seedlings was amplified by RT-PCR. All values were normalized against the expression level of the ACTIN2 gene. (0.11 MB TIF) [file pgen.1000817.s001.tif]

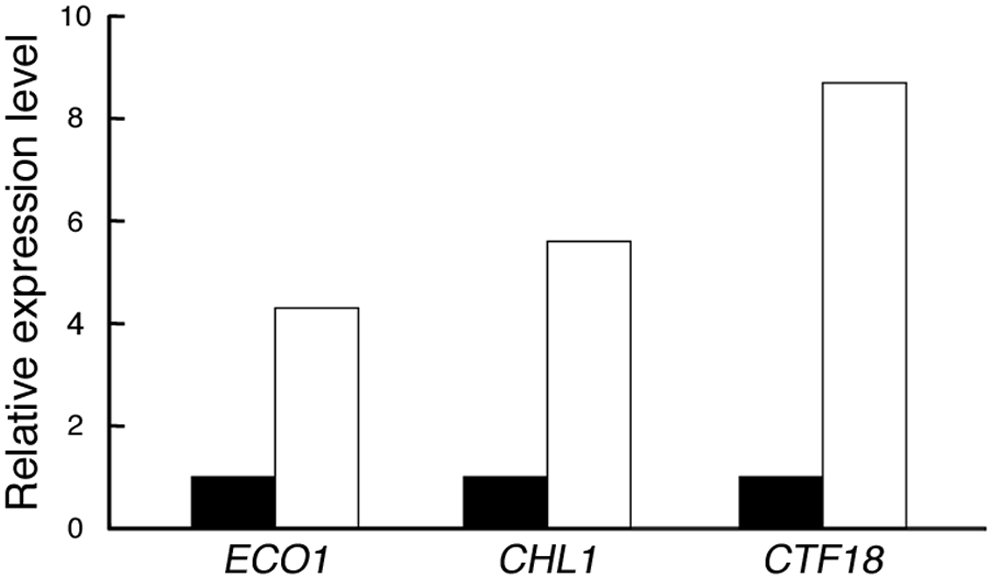

Supplement: Figure S2 — Upregulation of cohesion establishment genes in E2Fa-DPa-overexpressing plants. Relative expression level of cohesion establishment genes ECO1, CHL1, and CTF18 in wild-type (black) and E2Fa-DPa -overexpressing (white) plants. Data were imported from [25]. (0.07 MB TIF) [file pgen.1000817.s002.tif]

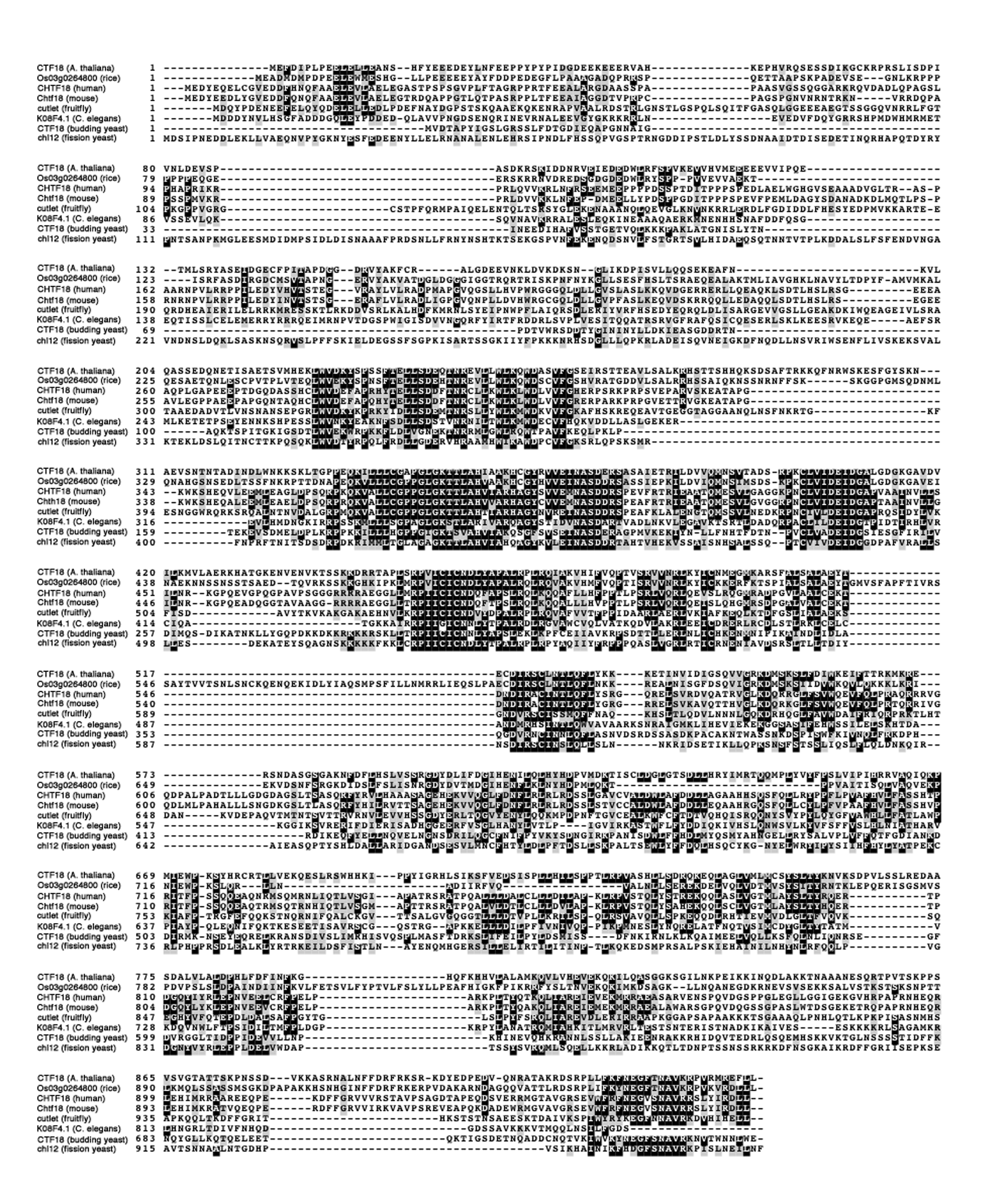

Supplement: Figure S3 — Conservation of the CTF18 protein in eukaryotes. Alignment of Arabidopsis CTF18 (ATCTF18) and its orthologous proteins: Os03g0264800 (rice), CHTF18 (NP_071375; human), Chtf18 (NP_663384; mouse), cutlet (NP_787969; fruitfly), K08F4.1 (NP_501841; C. elegans), CTF18 (NP_013795; budding yeast), and chl12 (NP_595200; fission yeast). Amino acid similarity between ATCTF18 and its orthologous proteins is 45% for rice, 30% for human, 30% for mouse, 29% for fruitfly, 27% for C. elegans, 26% for budding yeast, and 24% for fission yeast. (3.44 MB TIF) [file pgen.1000817.s003.tif]

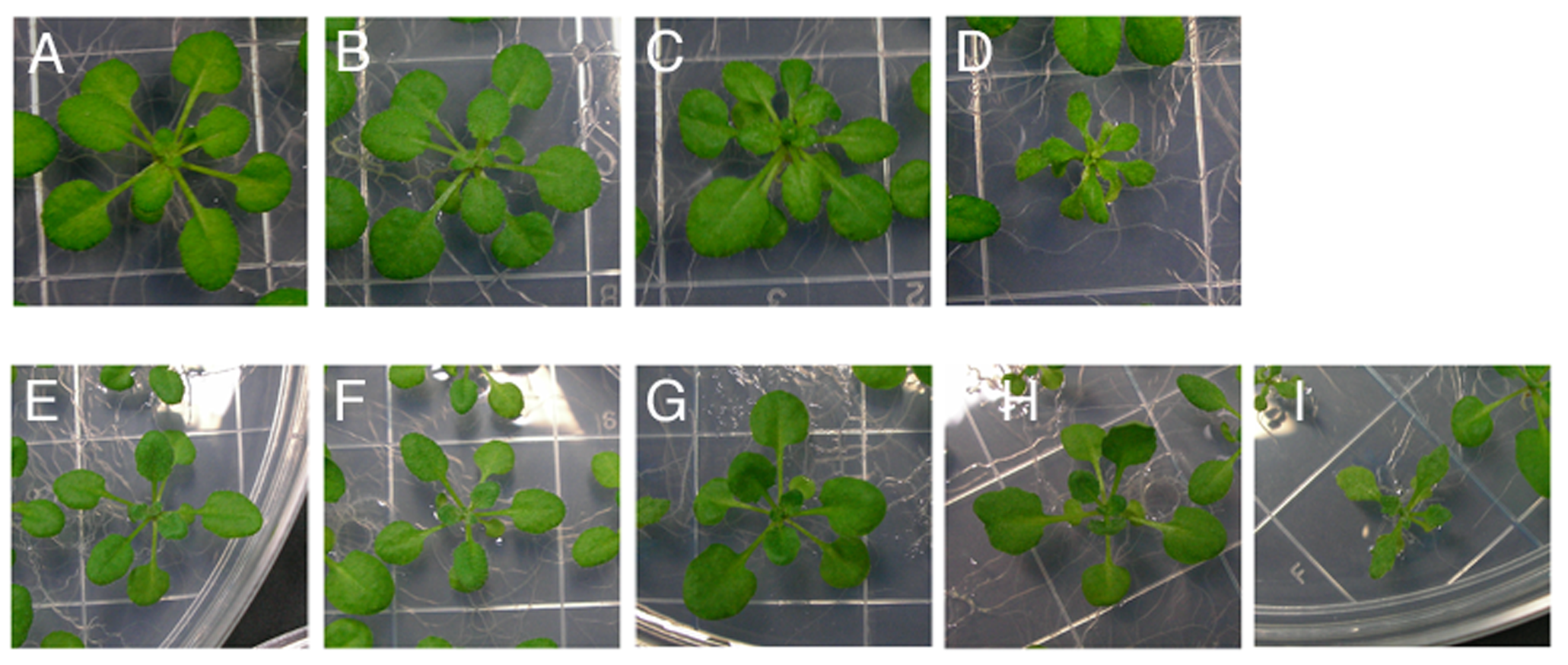

Supplement: Figure S4 — Genetic interaction between etg1 and ctf18 mutants on plant growth. (A-D) Seedling phenotypes of 21-day-old wild-type (Col-0) (A), etg1-2 (Col-0 background) (B), ctf18-2 (Col-0 background) (C), and etg1-2 ctf18-2 (D) plants. (E-I) Seedlings phenotype of 21-day-old wild-type (Col-0) (E), etg1-2 (Col-0 background) (F), wild-type (Nos-0) (G), ctf18-1 (Nos-0 background) (H), and etg1-2 ctf18-1 (I) plants. (2.33 MB TIF) [file pgen.1000817.s004.tif]

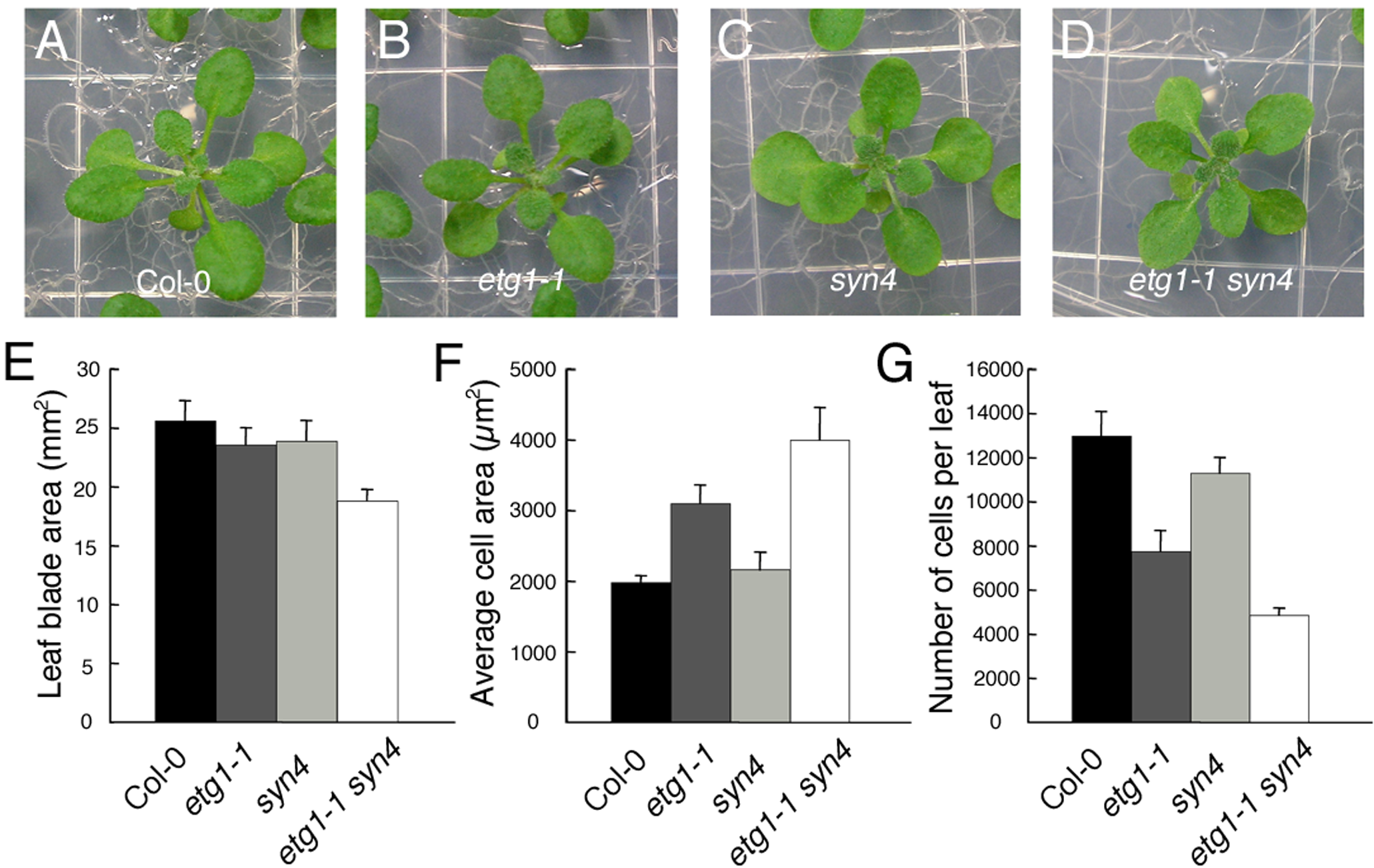

Supplement: Figure S5 — Genetic interaction between etg1 and cohesin mutant syn4. (A-D) Seedling phenotypes of 21-day-old wild-type (Col-0) (A), etg1-2 (Col-0 background) (B), syn4 (Col-0 background) (C), and etg1-2 syn4 (D) plants. (E-G) Leaf growth of the first leaf pair of 21-day-old wild-type (Col-0), etg1-1, syn4, and etg1-1 syn4 plants. Leaf blade area (E), epidermal cell size on the abaxial side of the leaf (F), and epidermal cell number on the abaxial side of the leaf (G). Data represent average ± SD (n = 5). (1.86 MB TIF) [file pgen.1000817.s005.tif]
