# Supplementary material for: The MCM-Binding Protein ETG1 Aids Sister Chromatid Cohesion Required for Postreplicative Homologous Recombination Repair
Source: PLoS Genet. 2010 Jan 15;6(1):e1000817. doi: 10.1371/journal.pgen.1000817 (PMC2806904; doi:10.1371/journal.pgen.1000817)
Supplement: Table S1 — Upregulated genes in etg1 compared with the wild-type genes (Col-0). (0.20 MB DOC) [file pgen.1000817.s006.doc]

**Table S1.** Upregulated genes in *etg1* compared with wild-type (Col-0)

| Fold change | | Gene code | Gene description | Phase^1^ | MSA^2^ | GO category |
| --- | --- | --- | --- | --- | --- | --- |
| *etg1-1* | *etg1-2* |  |  |  |  |  |
| 14.82 | 15.10 | At3g55920 | Peptidyl-prolyl cis-trans isomerase, putative |  |  | Protein folding |
| 6.91 | 5.92 | At5g60250 | Zinc finger (C3HC4-type ring finger) family protein |  | Y | Biological process unknown |
| 4.28 | 4.30 | At5g24280 | ATP binding protein |  |  | ATP binding |
| 4.19 | 3.99 | At5g23910 | Kinesin motor protein-related | M |  | Microtubule-based movement |
| 4.00 | 5.03 | At2g44450 | Glycosyl hydrolase family 1 protein |  |  | Carbohydrate metabolic process |
| 3.51 | 3.55 | At4g37490 | "CyclinB1;1 (CycB1;1)" |  |  | Regulation of progression through cell cycle |
| 3.39 | 3.15 | At4g02390 | Poly(ADP-ribose) polymerase (APP) |  |  | Protein amino acid ADP-ribosylation |
| 3.35 | 3.20 | At5g61070 | Histone deacetylase 18 (HDA18) |  |  | Chromatin remodeling |
| 3.32 | 3.39 | At3g44050 | Kinesin motor protein-related | M | Y | Microtubule-based movement |
| 3.23 | 2.97 | At5g48720 | XRI (X-RAY INDUCED TRANSCRIPT) |  |  | Biological process unknown |
| 3.07 | 3.59 | At3g02120 | Hydroxyproline-rich glycoprotein family protein | M | Y | Biological process unknown |
| 3.05 | 3.39 | At3g17680 | Unknown protein | M |  | Biological process unknown |
| 3.05 | 3.25 | At5g45700 | NLI interacting factor (NIF) family protein | M | Y | Phosphatase activity |
| 2.92 | 3.41 | At1g03660 | Similar to ankyrin repeat family protein |  |  | Biological process unknown |
| 2.89 | 2.66 | At3g27060 | TSO2 |  |  | DNA repair, DNA replication, regulation of progression through cell cycle |
| 2.80 | 2.56 | At3g23890 | TOPII (TOPOISOMERASE II) | M | Y | DNA metabolic process |
| 2.79 | 2.65 | At5g51600 | PLE (PLEIADE) | M |  | Cytokinesis by cell plate formation |
| 2.78 | 2.95 | At4g35620 | "CYCB2;2 (Cyclin B2;2)" | M | Y | Regulation of progression through cell cycle |
| 2.75 | 2.74 | At1g76540 | "CDKB2;1 (Cyclin-dependent kinase B2;1)" | M | Y | G2/M transition of mitotic cell cycle |
| 2.75 | 2.71 | At5g05180 | Unknown protein | M |  | Biological process unknown |
| 2.68 | 2.59 | At3g51740 | IMK2 (Inflorescence meristem receptor-like kinase 2) | M | Y | Protein amino acid phosphorylation |
| 2.68 | 2.47 | At5g55520 | Kinesin related protein | M |  | Biological process unknown |
| 2.66 | 2.94 | At2g24970 | Similar to Os01g0766400 |  |  | Biological process unknown |
| 2.66 | 2.94 | At3g22880 | ATDMC1 (RECA-LIKE GENE) |  |  | Meiosis |
| 2.65 | 2.54 | At3g51280 | Male sterility MS5, putative | M | Y | Biological process unknown |
| 2.65 | 2.47 | At4g05520 | ATEHD2 (EPS15 HOMOLOGY DOMAIN ) | M | Y | Endocytosis |
| 2.61 | 2.86 | At3g02640 | Unknown protein | M |  | Biological process unknown |
| 2.61 | 2.58 | At5g56580 | ATMKK6 (Arabidopsis NQK1) |  |  | Protein amino acid phosphorylation |
| 2.60 | 2.47 | At2g25880 | ATAUR2 (ATAURORA2) | M |  | Histone phosphorylation |
| 2.60 | 2.39 | At3g07800 | Thymidine kinase, putative |  |  | Anaerobic respiration, pyrimidine deoxyribonucleoside interconversion |
| 2.56 | 2.71 | At2g27970 | CKS2 (CDK-subunit 2) |  |  | Cell cycle |
| 2.56 | 2.50 | At5g17160 | Unknown protein | M |  | Biological process unknown |
| 2.55 | 2.66 | At4g05190 | ATK5 (Arabidopsis thaliana kinesin 5) | M | Y | Microtubule cytoskeleton organization and biogenesis, spindle assembly |
| 2.53 | 2.76 | At2g30360 | CIPK11 |  |  | Protein amino acid phosphorylation, signal transduction |
| 2.53 | 2.57 | At5g11510 | MYB3R-4 (c-myb-like transcription factor 3R-4) | M | Y | Cell cycle, regulation of transcription |
| 2.52 | 2.49 | At4g01730 | Zinc finger (DHHC type) family protein | M | Y | Biological process unknown |
| 2.51 | 2.51 | At1g76310 | "CYCB2;4 (CYCLIN B2;4)" | M | Y | Regulation of progression through cell cycle |
| 2.51 | 2.87 | At5g55180 | Glycosyl hydrolase family 17 protein | M | Y | Carbohydrate metabolic process |
| 2.47 | 2.60 | At1g08560 | KN (KNOLLE) | M | Y | Intracellular protein transport |
| 2.47 | 2.58 | At4g32830 | ATAUR1 (ATAURORA1) | M |  | Histone phosphorylation |
| 2.46 | 2.65 | At4g02800 | Unknown protein | M |  | Biological process unknown |
| 2.46 | 2.58 | At1g20930 | "CDKB2;2 (CYCLIN-DEPENDENT KINASE B2;2)" | M | Y | M phase of mitotic cell cycle |
| 2.44 | 2.61 | At2g26760 | "CYCB1;4" | M | Y | Regulation of progression through cell cycle |
| 2.44 | 2.30 | At5g60930 | Chromosome-associated kinesin | M | Y | Microtubule-based movement |
| 2.43 | 2.57 | At1g18370 | HINKEL (HIK) | M | Y | Microtubule-based movement |
| 2.43 | 2.74 | At2g37420 | Kinesin motor protein-related | M | Y | Microtubule-based movement |
| 2.42 | 2.60 | At5g15510 | Unknown protein | M |  | Biological process unknown |
| 2.42 | 2.83 | At4g15830 | Binding protein | M |  | Biological process unknown |
| 2.41 | 2.71 | At2g17620 | "CYCB2;1 (CYCLIN B2;1)" | M | Y | Regulation of progression through cell cycle |
| 2.37 | 2.60 | At3g25980 | Mitotic spindle checkpoint protein, putative (MAD2) | M | Y | Mitotic cell cycle spindle assembly checkpoint |
| 2.37 | 2.58 | At3g52110 | Hypothetical protein | M |  | Biological process unknown |
| 2.36 | 2.29 | At3g23670 | PAKRP1L | M | Y | Microtubule-based movement |
| 2.36 | 2.46 | At1g28290 | Pollen Ole e 1 allergen and extensin family protein | M |  | Biological process unknown |
| 2.35 | 2.32 | At1g44110 | "CYCA1;1 (CYCLIN A1;1);" | M |  | Regulation of progression through cell cycle |
| 2.34 | 2.38 | At3g15560 | Unknown protein | M |  | Biological process unknown |
| 2.33 | 2.19 | At1g31335 | Unknown protein |  |  | Biological process unknown |
| 2.33 | 2.22 | At3g06030 | Arabidopsis NPK1-related protein kinase 3 (ANP3) |  | Y | Protein amino acid phosphorylation |
| 2.32 | 2.16 | At4g22960 | Unknown protein |  |  | Biological process unknown |
| 2.32 | 1.89 | At3g60840 | Microtubule associated protein (MAP65/ASE1) family protein | M |  | Microtubule associated |
| 2.31 | 2.54 | At5g62550 | Unknown protein | M |  | Biological process unknown |
| 2.30 | 2.57 | At1g23790 | Unknown protein | M |  | Biological process unknown |
| 2.30 | 2.53 | At3g20150 | Kinesin motor family protein | M | Y | Microtubule-based movement |
| 2.29 | 2.12 | At2g42110 | Unknown protein | M |  | Biological process unknown |
| 2.29 | 2.26 | At5g16250 | Unknown protein | M |  | Biological process unknown |
| 2.29 | 2.34 | At5g13840 | ccs52B | M | Y | Signal transduction |
| 2.29 | 2.71 | At1g34355 | Forkhead-associated domain-containing protein | M | Y | Biological process unknown |
| 2.29 | 2.19 | At4g28230 | Unknown protein | M |  | Biological process unknown |
| 2.29 | 2.10 | At1g02690 | Importin alpha-2 subunit, putative | M | Y | Intracellular protein transport |
| 2.28 | 2.29 | At1g02730 | Cellulose synthase-like D5 (ATCSLD5) | M | Y | Cellulose synthase activity |
| 2.28 | 2.30 | At5g66230 | Similar to sugar transporter superfamily | M | Y | Biological process unknown |
| 2.27 | 3.15 | At3g42725 | Unknown protein | M |  | Biological process unknown |
| 2.26 | 2.23 | At1g50240 | FUSED (FU) |  | Y | Cellularization of the embryo sac, cytokinesis by cell plate formation |
| 2.26 | 2.00 | At1g63100 | SCARECROW transcription factor | M | Y | Regulation of transcription |
| 2.26 | 2.18 | At1g18250 | Arabidopsis thaumatin-like protein 1 (ATLP-1) | M | Y | Response to other organism |
| 2.25 | 2.08 | At1g72250 | Kinesin motor protein-related | M | Y | Microtubule-based movement |
| 2.24 | 2.13 | At4g31805 | WRKY family transcription factor |  | Y | Regulation of transcription |
| 2.24 | 2.04 | At1g03780 | Targeting protein-related | M | Y | Biological process unknown |
| 2.23 | 2.40 | At5g67270 | Microtuble-end-binding protein 1 (ATEB1C) | M | Y | Cortical cytoskeleton organization and biogenesis |
| 2.23 | 2.91 | At2g18600 | RUB1-conjugating enzyme, putative |  | Y | Protein modification process, ubiquitin cycle |
| 2.21 | 1.99 | At3g57860 | UVB-insensitive 4-like |  | Y | Biological process unknown |
| 2.16 | 2.31 | At5g48310 | Unknown protein | M |  | Biological process unknown |
| 2.12 | 2.06 | At1g50490 | Ubiquitin-conjugating enzyme 20 (UBC20) | M | Y | Cell proliferation |
| 2.12 | 1.87 | At3g17360 | Phragmoplast orienting kinesin 1 (POK1) | M | Y | Microtubule-based movement |
| 2.10 | 2.32 | At5g47500 | Pectinesterase family protein | M | Y | Cell wall modification |
| 2.10 | 2.14 | At2g28620 | Kinesin motor protein-related | M | Y | Microtubule-based movement |
| 2.09 | 1.96 | At2g22610 | Kinesin motor protein-related | M | Y | Microtubule-based movement |
| 2.07 | 2.03 | At4g21820 | Calmodulin-binding family protein | M | Y | Calmodulin binding |
| 2.07 | 1.98 | At1g66620 | Seven in absentia (SINA) protein, putative |  |  | Multicellular organismal development, ubiquitin-dependent protein catabolic process |
| 2.07 | 1.90 | At1g23000 | Heavy-metal-associated domain-containing protein |  | Y | Metal ion transport |
| 2.07 | 2.33 | At4g11080 | High mobility group (HMG1/2) family protein | M | Y | Regulation of transcription |
| 2.05 | 1.95 | At1g73620 | Thaumatin-like protein, putative |  |  | Response to other organism |
| 2.03 | 2.21 | At1g78430 | Tropomyosin-related |  | Y | Biological process unknown |
| 2.02 | 2.10 | At5g01910 | Unknown protein | M |  | Biological process unknown |
| 2.03 | 1.87 | At2g33560 | Spindle checkpoint protein-related | M | Y | Biological process unknown |
| 2.02 | 2.05 | At3g55660 | ATROPGEF6/ROPGEF6 (Kinase partner protein-like) | M |  | Biological process unknown |
| 2.00 | 1.79 | At4g14330 | Phragmoplast-associated kinesin-related protein 2 (PAKRP2) | M | Y | Microtubule-based movement |
| 1.99 | 1.78 | At5g40840 | Sister chromatid cohesion 1 (SCC1) protein homolog 2 (SYN2) |  |  | Mitosis |
| 1.98 | 2.12 | At5g37010 | Unknown protein | M |  | N-terminal protein myristoylation |
| 1.98 | 1.74 | At5g27550 | Kinesin motor protein-related | M | Y | Microtubule-based movement |
| 1.98 | 2.14 | At3g14190 | Unknown protein | M |  | Biological process unknown |
| 1.96 | 2.04 | At1g10780 | F-box family protein | M |  | Biological process unknown |
| 1.94 | 2.16 | At1g59540 | Kinesin-like protein | M | Y | Microtubule-based movement |
| 1.94 | 2.02 | At3g58650 | Unknown protein | M |  | Biological process unknown |
| 1.92 | 1.99 | At1g53140 | Dynamin family protein | M | Y | Biological process unknown |
| 1.92 | 1.67 | At3g10310 | Kinesin motor protein-related | M | Y | Microtubule-based movement |
| 1.91 | 1.85 | At1g49870 | Unknown protein | M |  | Biological process unknown |
| 1.91 | 1.90 | At4g24610 | Unknown protein |  |  | Biological process unknown |
| 1.91 | 1.77 | At5g03780 | TRF-like 10 (TRFL10) |  |  | Response to salicylic acid stimulus |
| 1.90 | 1.69 | At2g36200 | Kinesin motor protein-related |  | Y | Microtubule-based movement |
| 1.90 | 1.65 | At1g57820 | Variant in methylation 1 (VIM1) |  | Y | Regulation of transcription, DNA-dependent, centric heterochromatin formation, DNA methylation on cytosine |
| 1.89 | 1.83 | At4g03100 | Rac GTPase activating protein, putative | G2 |  | Signal transduction |
| 1.88 | 1.98 | At3g12870 | Unknown protein | M |  | Biological process unknown |
| 1.87 | 1.79 | At3g27330 | Zinc finger (C3HC4-type RING finger) family protein | M | Y | Protein ubiquitination |
| 1.87 | 1.90 | At4g33400 | Defective embryo and meristems protein-related (DEM) | M | Y | N-terminal protein myristoylation |
| 1.86 | 1.98 | At4g21270 | ATK1 (Arabidopsis thaliana kinesin 1) |  | Y | Anastral spindle assembly involved in male meiosis |
| 1.85 | 1.83 | At4g28950 | ARAC7/ATROP9/RAC7/ROP9 | S | Y | Small GTPase mediated signal transduction |
| 1.85 | 2.08 | At2g25060 | Plastocyanin-like domain-containing protein | M | Y | Electron transport |
| 1.84 | 1.52 | At1g30160 | Unknown protein |  |  | Biological process unknown |
| 1.84 | 1.97 | At4g31840 | Plastocyanin-like domain-containing protein | M | Y | Electron transport |
| 1.84 | 1.87 | At5g03870 | Glutaredoxin family protein | M | Y | N-terminal protein myristoylation |
| 1.84 | 2.61 | At3g15550 | Hypothetical protein |  |  | Biological process unknown |
| 1.84 | 2.06 | At2g07170 | Similar to TORTIFOLIA 1 (TOR1) | M |  | Biological process unknown |
| 1.82 | 1.73 | At5g63920 | DNA topoisomerase III alpha, putative |  | Y | DNA topological change, DNA unwinding during replication |
| 1.80 | 2.15 | At5g55830 | Lectin protein kinase, putative | M | Y | Protein amino acid phosphorylation |
| 1.79 | 1.74 | AT3G03130 | Unknown protein | M | Y | Biological process unknown |
| 1.79 | 1.71 | AT5G62410 | SMC2 (STRUCTURAL MAINTENANCE OF CHROMOSOMES 2) |  | Y | DNA metabolic process, chromosome organization |
| 1.78 | 1.65 | AT1G16070 | AtTLP8 (TUBBY LIKE PROTEIN 8) |  | Y | Regulation of transcription |
| 1.77 | 1.84 | AT5G64060 | ANAC103 (Arabidopsis NAC domain containing protein 103) |  |  | Transcription factor activity |
| 1.77 | 1.74 | AT5G60150 | Unknown protein |  | Y | Biological process unknown |
| 1.77 | 1.85 | AT2G26180 | IQD6 (IQ-domain 6) |  |  | Biological process unknown |
| 1.77 | 1.55 | AT3G63480 | Kinesin heavy chain, putative |  |  | Microtubule-based movement |
| 1.75 | 1.64 | AT3G56100 | MRLK (MERISTEMATIC RECEPTOR-LIKE KINASE) |  | Y | Protein amino acid phosphorylation, Transmembrane receptor protein tyrosine kinase signaling pathway |
| 1.75 | 1.76 | AT3G11520 | "CYCB1;3 (CYCLIN B1;3)" | M | Y | Cell cycle, Regulation of cell cycle |
| 1.75 | 1.87 | AT2G29890 | VLN1 (VILLIN-LIKE 1) |  |  | Actin filament organization, Cytoskeleton organization, Negative regulation of actin filament depolymerization |
| 1.74 | 1.80 | AT5G44560 | VPS2.2 | M | Y | Vesicle-mediated transport |
| 1.73 | 1.78 | AT4G39630 | Unknown protein | S | Y | Biological process unknown |
| 1.73 | 2.04 | AT2G44190 | Unknown protein | M | Y | Biological process unknown |
| 1.73 | 1.79 | AT5G01370 | Unknown protein | G1 |  | Biological process unknown |
| 1.71 | 1.69 | AT2G37390 | Heavy-metal-associated domain-containing protein |  |  | Metal ion transport |
| 1.68 | 1.64 | AT5G05940 | ATROPGEF5/ROPGEF5 (ROP GUANINE NUCLEOTIDE EXCHANGE FACTOR 5) | M | Y | Rho guanyl-nucleotide exchange factor activity |
| 1.68 | 1.74 | AT1G11600 | CYP77B1 (cytochrome P450, family 77, subfamily B, polypeptide 1) |  |  | Oxygen binding |
| 1.66 | 1.33 | AT2G32590 | Similar to Barren |  |  | Mitosis, Mitotic cell cycle |
| 1.65 | 1.75 | AT5G33300 | Chromosome-associated kinesin-related protein |  |  | Biological process unknown |
| 1.65 | 1.55 | AT1G17480 | IQD7 (IQ-domain 7) |  |  | Biological process unknown |
| 1.65 | 1.40 | AT1G02670 | DNA repair protein, putative |  | Y | DNA repair |
| 1.65 | 1.63 | AT3G60900 | FLA10 (fasciclin-like arabinogalactan-protein 10) |  | Y | Biological process unknown |
| 1.65 | 1.51 | AT3G01330 | DEL3 (DP-E2F-like protein 3) |  |  | Regulation of transcription, DNA-dependent |
| 1.65 | 1.51 | AT2G47500 | Microtubule motor protein | M |  | Microtubule-based movement |
| 1.65 | 1.48 | AT2G45460 | Forkhead-associated domain-containing protein |  |  | Regulation of transcription, DNA-dependent |
| 1.64 | 1.85 | AT2G29570 | PCNA2 (PROLIFERATING CELL NUCLEAR ANTIGEN 2) |  |  | Regulation of DNA replication, Error-prone postreplication DNA repair |
| 1.64 | 1.84 | AT2G34920 | EDA18 (embryo sac development arrest 18) | M | Y | Embryo sac development |
| 1.64 | 1.45 | AT1G76740 | Unknown protein | M |  | Biological process unknown |
| 1.63 | 1.46 | AT1G09815 | POLD4 (polymerase delta 4) |  | Y | DNA replication |
| 1.62 | 1.73 | AT1G50240 | FU (FUSED) |  | Y | Cellularization of the embryo sac, Cytokinesis by cell plate formation |
| 1.61 | 1.61 | AT1G21810 | Similar to myosin heavy chain-related protein |  |  | Biological process unknown |
| 1.61 | 1.77 | AT4G26660 | Similar to kinesin related protein | M |  | Biological process unknown |
| 1.59 | 1.51 | AT5G09220 | AAP2 (AMINO ACID PERMEASE 2) |  |  | Acidic amino acid transport, Amino acid transport, Neutral amino acid transport |
| 1.58 | 1.41 | AT5G66130 | ATRAD17 (RADIATION SENSITIVE 17) |  |  | DNA repair, Cell cycle |
| 1.58 | 1.58 | AT5G60880 | Unknown protein |  |  | Biological process unknown |
| 1.58 | 1.57 | AT1G54960 | ANP2 (Arabidopsis NPK1-related protein kinase 2) |  |  | Cytokinesis |
| 1.57 | 1.37 | AT5G52220 | Hypothetical protein | S | Y | Biological process unknown |
| 1.57 | 1.52 | AT3G19590 | Mitotic checkpoint protein, putative | M |  | Biological process unknown |
| 1.56 | 1.68 | AT2G40670 | ARR16 (arabidopsis response regulator 16) |  |  | Response to cytokinin stimulus, Cytokinin mediated signaling |
| 1.56 | 1.43 | AT3G57060 | Similar to binding protein |  |  | Chromosome condensation |
| 1.56 | 1.41 | AT1G47870 | E2FC (ARABIDOPSIS HOMOLOG OF E2F C) |  |  | DNA endoreduplication, cell morphogenesis, Cell division, Negative regulation of cell division |
| 1.56 | 1.66 | AT5G48460 | Fimbrin-like protein, putative | M | Y | Biological process unknown |
| 1.54 | 1.90 | AT1G16330 | "CYCB3;1 (CYCLIN B3;1)" | M |  | Regulation of cell cycle |
| 1.54 | 1.40 | AT5G25490 | Zinc finger (Ran-binding) family protein |  |  | Biological process unknown |
| 1.52 | 1.44 | AT1G36180 | ACC2 (ACETYL-COA CARBOXYLASE 2) |  | Y | Metabolic process |
| 1.52 | 1.41 | AT2G31320 | Poly (ADP-ribose) polymerase, putative |  | Y | DNA repair, Protein amino acid ADP-ribosylation |
| 1.52 | 1.38 | AT2G01120 | ATORC4/ORC4 (ORIGIN RECOGNITION COMPLEX SUBUNIT 4) |  |  | DNA replication |
| 1.52 | 1.36 | AT4G17000 | Unknown protein | M | Y | Biological process unknown |
| 1.51 | 1.36 | AT2G38160 | Similar to proline-rich family protein | M |  | Biological process unknown |
| 1.50 | 1.39 | AT1G31280 | AGO2 (ARGONAUTE 2) |  |  | Biological process unknown |
| 1.49 | 1.38 | AT1G14260 | Zinc finger (C3HC4-type RING finger) family protein |  |  | Protein binding, Zinc ion binding |
| 1.48 | 1.35 | AT5G50890 | Similar to lipase class 3 family protein | M |  | Biological process unknown |
| 1.47 | 1.36 | AT1G63160 | Replication factor C 40 kDa, putative |  |  | DNA replication |
| 1.47 | 1.38 | AT2G45780 | Unknown protein |  |  | Biological process unknown |
| 1.44 | 1.38 | AT5G61460 | MIM (HYPERSENSITIVE TO MMS, IRRADIATION AND MMC) |  |  | Chromosome segregation |
| 1.44 | 1.50 | AT5G45400 | Replication protein, putative |  | Y | DNA replication |
| 1.43 | 1.62 | AT3G10880 | Unknown protein | G2 |  | Biological process unknown |
| 1.42 | 1.39 | AT5G63950 | CHR24 (chromatin remodeling 24) |  |  | Response to cadmium ion |
| 1.42 | 1.60 | AT5G50930 | Hypothetical protein |  | Y | Biological process unknown |
| 1.42 | 1.43 | AT1G50010 | TUA2 (tubulin alpha-2 chain) |  |  | Microtubule-based process |
| 1.41 | 1.43 | AT3G50070 | "CYCD3;3 (CYCLIN D3;3)" |  | Y | Regulation of cell proliferation, Regulation of cell cycle |
| 1.38 | 1.31 | AT4G08685 | SAH7 |  |  | Biological process unknown |
| 1.38 | 1.58 | AT1G07880 | ATMPK13 (ARABIDOPSIS THALIANA MAP KINASE 13) | M |  | Signal transduction |
| 1.36 | 1.42 | AT1G35780 | Unknown protein |  |  | Biological process unknown |
| 1.35 | 1.66 | AT1G34770 | MAGE-8 antigen-related protein |  |  | Biological process unknown |
| 1.34 | 1.39 | AT5G56780 | Unknown protein |  |  | Biological process unknown |
| 1.34 | 1.63 | AT4G15093 | Catalytic LigB subunit of aromatic ring-opening dioxygenase family |  |  | Cellular aromatic compound metabolic process |
| 1.33 | 1.30 | AT3G25905 | CLE27 (CLAVATA3/ESR-RELATED 27) |  |  | Signal transduction |
| 1.33 | 1.41 | AT5G11460 | Senescence-associated protein |  | Y | Biological process unknown |
| 1.32 | 1.45 | AT5G48020 | Hypothetical protein |  |  | Biological process unknown |
| 1.31 | 1.33 | AT2G01580 | Unknown protein |  |  | Biological process unknown |

^1^Refers to the peak of expression in the *Arabidopsis* cell cycle as defined by Menges et al. (2003)

^2^Refers to the presence of a mitosis-specific activator *cis*-acting element (MSA) within the first 1kb region upstream of the translation start
