# Supplementary material for: The MCM-Binding Protein ETG1 Aids Sister Chromatid Cohesion Required for Postreplicative Homologous Recombination Repair
Source: PLoS Genet. 2010 Jan 15;6(1):e1000817. doi: 10.1371/journal.pgen.1000817 (PMC2806904; doi:10.1371/journal.pgen.1000817)
Supplement: Table S2 — Downregulated genes in etg1 compared with the wild-type genes (Col-0). (0.06 MB DOC) [file pgen.1000817.s007.doc]

**Table S2.** Downregulated genes in *etg1* compared with wild-type

| Fold change | | | | Gene code | Gene description | Phase^1^ | MSA^2^ | | GO category |
| --- | --- | --- | --- | --- | --- | --- | --- | --- | --- |
| *etg1-1* | | *etg1-2* | |  |  |  |  | |  |
| 0.69 | 0.64 | | AT1G04800 | | Glycine-rich protein |  |  | N-terminal protein myristoylation | |
| 0.69 | 0.66 | | AT4G28085 | | Unknown protein |  |  | Biological process unknown | |
| 0.68 | 0.59 | | AT3G53720 | | ATCHX20 (CATION/H+ EXCHANGER 20) |  | Y | Cation transport, Cellular potassium ion homeostasis, Water homeostasis | |
| 0.67 | 0.60 | | AT4G38810 | | Calcium-binding EF hand family protein |  |  | Calcium ion binding | |
| 0.66 | 0.62 | | AT3G11930 | | Universal stress protein (USP) family protein |  | Y | Response to stress | |
| 0.66 | 0.41 | | AT2G05380 | | GRP3S (GLYCINE-RICH PROTEIN 3 SHORT ISOFORM) |  |  | Biological process unknown | |
| 0.64 | 0.69 | | AT3G24140 | | FMA (FAMA) |  |  | Positive regulation of cell differentiation, Positive regulation of transcription, Guard cell differentiation, Negative regulation of cell division | |
| 0.64 | 0.69 | | AT4G37580 | | COP3 (CONSTITUTIVE PHOTOMORPHOGENIC 3) |  | Y | Metabolic process, Photomorphogenesis, Unidimensional cell growth, Response to ethylene stimulus, auxin mediated signaling pathway | |
| 0.63 | 0.38 | | AT2G29490 | | ATGSTU1 (GLUTATHIONE S-TRANSFERASE TAU 1) |  |  | Toxin catabolic process | |
| 0.62 | 0.62 | | AT2G16380 | | SEC14 cytosolic factor family protein / phosphoglyceride transfer family protein |  | Y | Transport | |
| 0.61 | 0.55 | | AT4G12430 | | Trehalose-6-phosphate phosphatase, putative |  |  | Trehalose biosynthetic process | |
| 0.60 | 0.53 | | AT1G70530 | | Protein kinase family protein |  |  | Protein amino acid phosphorylation | |
| 0.59 | 0.56 | | AT1G08810 | | ATMYB60 (MYB DOMAIN PROTEIN 60) | M |  | Response to water deprivation, Response to light stimulus, Response to salt stress, Regulation of transcription, DNA-dependent, Response to abscisic acid stimulus, Response to auxin stimulus, response to ethylene stimulus, Response to gibberellin stimulus, Response to jasmonic acid stimulus, Response to salicylic acid stimulus, Response to cadmium ion, Stomatal movement | |
| 0.55 | 0.23 | | AT3G28740 | | CYP81D1 |  |  | Defense response to insect | |
| 0.54 | 0.46 | | AT3G48740 | | Nodulin MtN3 family protein |  | Y | Biological process unknown | |
| 0.54 | 0.53 | | AT1G75880 | | Similar to family II extracellular lipase 2 (EXL2) |  |  | Sexual reproduction | |
| 0.53 | 0.56 | | AT3G60520 | | Zinc ion binding |  | Y | Biological process unknown | |
| 0.53 | 0.46 | | AT1G23130 | | Bet v I allergen family protein |  |  | Defense response, Response to biotic stimulus | |
| 0.50 | 0.42 | | AT1G67860 | | Unknown protein |  |  | Biological process unknown | |
| 0.42 | 0.37 | | AT2G40960 | | Nucleic acid binding |  | Y | Biological process unknown | |
| 0.38 | 0.41 | | AT5G35490 | | Unknown protein |  |  | Biological process unknown | |
| 0.32 | 0.25 | | AT3G14210 | | ESM1 (EPITHIOSPECIFIER MODIFIER 1) |  |  | Response to cold, Response to insect, Glucosinolate catabolic process | |
| 0.28 | 0.39 | | AT3G30720 | | Unknown protein |  |  | Biological process unknown | |

^1^Refers to the peak of expression in the *Arabidopsis* cell cycle as defined by Menges et al. (2003)

^2^Refers to the presence of a mitosis-specific activator *cis*-acting element (MSA) within the first 1kb region upstream of the translation start
